# *FKS1/2*-variant independent mechanisms underlying the emergence of resistance in echinocandin-refractory *Candida auris* infections

**DOI:** 10.1038/s41467-026-76954-x

**Published:** 2026-10-15

**Authors:** Hugh Gifford, Arnab Pradhan, Ben Caswall, Sarah E. Murphy, Ian Leaves, Matt B. Edmondson, Tanmoy Chakraborty, Duncan Wilson, Surabhi K. Taori, Neil A. R. Gow, Rhys A. Farrer, Alistair J. P. Brown, Tihana Bicanic

**Affiliations:** 1 MRC Center for Medical Mycology at the University of Exeter, Geoffrey Pope Building, Stocker Road, Exeter, United Kingdom, EX4 4QD UK; 2 Institute of Infection & Immunity, St George’s School of Health and Medical Sciences, City St George’s University London, United Kingdom, SW17 0RE UK; 3 King’s College Hospital, Denmark Hill, London, SE5 9RS UK

**Keywords:** Fungal genomics, Fungal evolution, Antifungal agents, Antimicrobial resistance

## Abstract

The emerging fungus *Candida auris* is a drug resistant global public health threat and WHO critical priority pathogen. Recommended first-line invasive candidiasis treatment is echinocandin monotherapy, but *C. auris* can develop on-treatment resistance via *FKS1/2* gene mutations and additional, previously unexplained mechanisms. To better understand echinocandin failure in *C. auris*, we sequenced the genomes of echinocandin refractory *FKS1/2* wild-type *C. auris* serial isolates from two critically unwell patients in London, UK. Population analysis profiling revealed echinocandin heteroresistance, and in vitro culture of clinical isolates at supra-MIC concentrations of anidulafungin (8 μg/ml) exhibited morphotypic heterogeneity. Small colony variants (SCVs) and large colony variants (LCVs) showed elevated MICs with polyploidy (to 4n and above) alongside adaptive changes in cell wall β−1,3-glucan content. LCVs contained significantly more mutations in calcineurin-related stress tolerance pathway gene *CRZ1* compared to clinical parents and SCVs, associated with further increases in MIC. These findings indicate progressive step-wise accrual of adaptation to echinocandins, including genomic instability, alterations in stress tolerance pathways, and cell wall remodelling, paving the way for resistance emergence.

## Abstract (web summary)

Human fungal pathogen *Candida auris* demonstrates antifungal drug resistance emergence. Authors present their study showing that progressive step-wise adaptation results from genomic instability and polyploidy, followed by escape mutation in stress-tolerance pathways and cell wall remodelling.

## Introduction

*Candida auris*, also named *Candidozyma auris*^1^, is a World Health Organization critical priority human fungal pathogen^2^. This fungus causes invasive infections (candidiasis) with an associated mortality of up to 45%^3^, and propensity for resistance to all key antifungal classes: azoles, echinocandins, and polyenes^4,5^. After description in Japan in 2008^6^, six clades have emerged near-simultaneously across the world^7,8^. *C. auris* colonises the skin, particularly of patients who are critically ill or in long-term ventilation facilities^9^. Furthermore, *C. auris* is challenging to eradicate from hospital environments owing to its persistence on abiotic surfaces, such as contaminated surfaces and hospital equipment acting as fomites, resulting in inter-patient spread within and between hospitals^10–13^. Colonisation and infection rates continue to rise, with detection now reported across all seven continents, including Antarctica^14–20^. A recent surge in UK cases has prompted the inclusion of *C. auris* in the list of notifiable organisms, updates to guidelines for healthcare settings, and the declaration of a national public health incident in 2025^21,22^.

As the majority of *C. auris* isolates are resistant to fluconazole ( > 97% for clades I and III) and a high proportion to amphotericin B (47% for clade I)^12,23^, echinocandin monotherapy is currently the initial treatment recommended for invasive infections^5,24,25^. Initially, detection of echinocandin resistance was low across clades I-IV (0-9%)^23^, but cases continue to emerge^15^. Echinocandins target the essential cell wall enzyme β-glucan synthase, encoded by *FKS1* and *FKS2* in *C. auris*^26^. Resistance is typically defined as growth above a minimum inhibitory concentration (MIC) using epidemiologically derived or clinical breakpoints that correlate with clinical outcomes^27,28^. Typical resistance is thought to arise primarily through mutations in *FKS1* hot-spots^29–34^ that lead to treatment failure in vivo, as demonstrated in murine candidiasis models^35,36^. Several clinical case studies have shown resistance arising due to *FKS1* mutations: in a five isolate series from a single patient over 1 year, only the terminal isolate contained the F635Y *FKS1* variant and was echinocandin resistant^37^. In a nineteen isolate series from a single patient over 72 days, F639Y/F635C variants emerged alongside resistance to four classes of antifungal (pan-resistance) with further mutations in genes related to azole, polyene, and flucytosine resistance^38^.

Echinocandin resistance can also be caused by *FKS1/2*-independent mechanisms that either promote resistance directly or lead to *FKS* mutations. In four cases of urinary infection, *FKS1* mutation was necessary for echinocandin resistance, but other mutations related to cell wall stress and DNA repair/chromatin remodelling were present in clinical isolates with elevated MICs^39^. In vitro micro-evolution experiments have shown additional mutations outside *FKS1* hot-spots, including in *ERG3*, associated with echinocandin resistance^40,41^. Large-scale genome-wide association studies have suggested that mutations in other cell wall-related genes (*IFF4*, *FCR1,* and *GWT1*) may promote echinocandin resistance^42^. In *Candida albicans*^43^ (and other fungal pathogens^44,45^), aneuploidy and copy number variation can also lead to echinocandin resistance. Such structural variation has not yet been described in *C. auris* with respect to echinocandin resistance^46^. Furthermore, the development of secondary echinocandin resistance, and even pan-resistance to azoles, polyenes, echinocandins, and flucytosine, has been reported in isolates from patients receiving treatment for clinically refractory infection, described in as many as 3% of clade I isolates^15,23,38,47,48^. The step-wise mechanisms underlying the emergence of echinocandin resistance remain poorly understood and are urgent questions for *C. auris* therapy in light of such limited options^46,49^.

In addition to standard antifungal drug resistance, heteroresistance and tolerance phenomena can contribute to population heterogeneity, stress adaptation, and treatment failure in infections caused by other pathogenic *Candida* species^50^, including *Candida parapsilosis*^51^, *Candida glabrata* (*Nakaseomyces glabratus*)^52^ and *C. albicans*^53^. Heteroresistance describes a small intrinsically resistant sub-population of cells ( < 1%) that is selected for and expands under drug pressure^50,51,54^. Tolerance represents the ability of a larger sub-population of cells (10-50%) within an isogenic, drug-susceptible population (using MIC) to survive and grow slowly ( > 24 h) at concentrations above the MIC^50^. In other pathogenic *Candida* species, echinocandin tolerance has been shown to arise via increased chitin synthesis and cell wall remodeling triggered by protein kinase C (PKC), Hog1, Hsp90, and calcineurin signalling^55,56^. In *C. auris*, echinocandin exposure in vitro can lead to the increased expression of genes involved in cell wall synthesis and remodelling including genes encoding chitin synthases, cell wall adhesin Als5 and the drug efflux pump Cdr1^26,57,58^. These transcriptional changes correlate with echinocandin-induced adaptations such as cellular adhesion, aggregation, and biofilm formation^57–59^.

Here, we explore underlying mechanisms associated with the emergence of echinocandin refractory infection in two critically ill patients with *C. auris* candidaemia. Our data provide evidence for the sequential accumulation of a series of physiological and genetic adaptations leading to the emergence of resistance and echinocandin treatment failure.

## Results

### *FKS1* mutations and aneuploidy are insufficient to account for echinocandin refractory *C. auris* infection

**Fig. 1:**
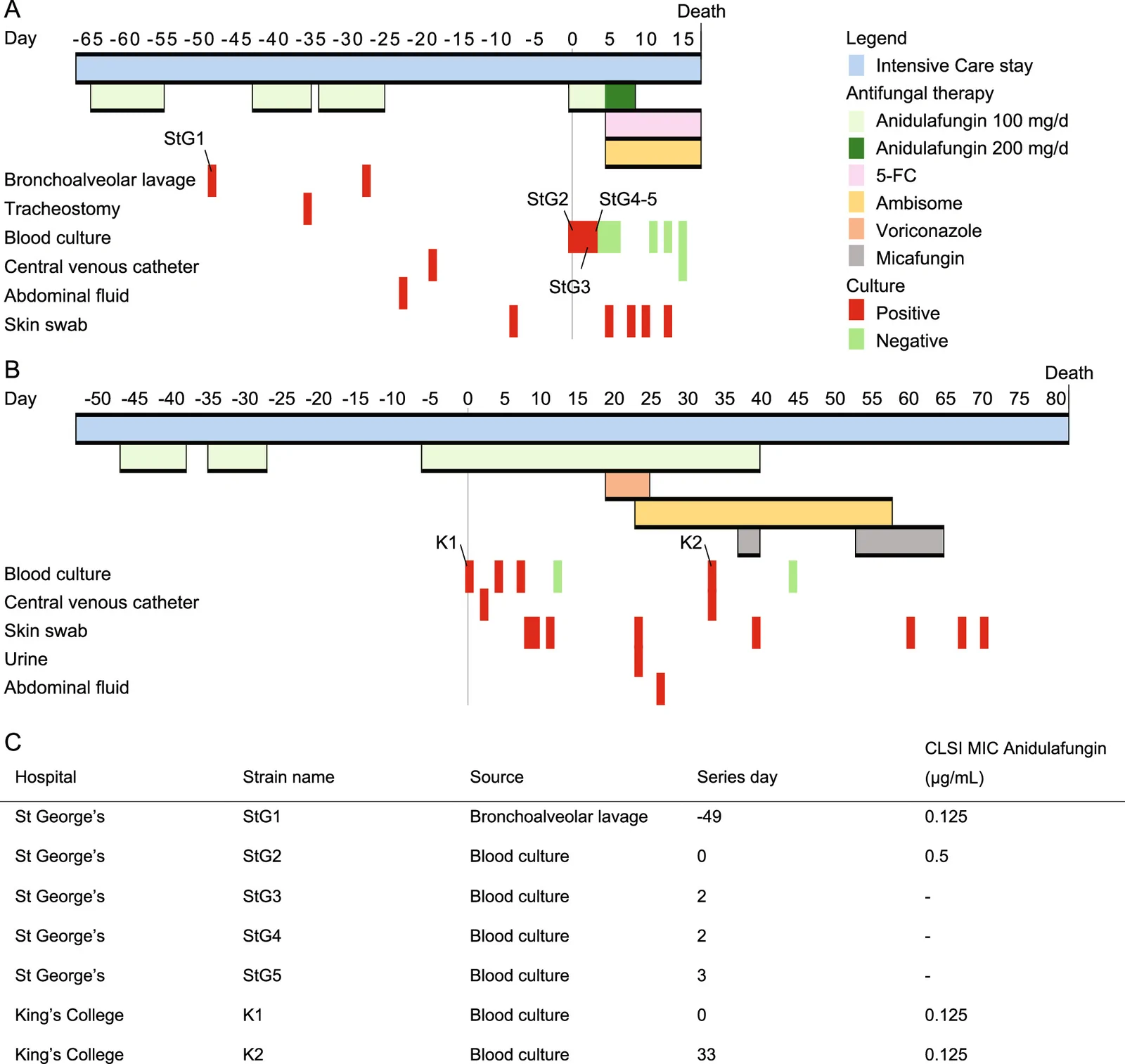
Clinical case series for patients suffering from *C. auris* candidaemia during antifungal therapy, both pre- and post-clinical isolate collection. **A** St George’s Hospital case series (isolates StG1-5), beginning at day −67 from first blood culture isolation (day 0), and ending at death on day 17. **B** King’s College Hospital case series (isolates K1-2), beginning at day −53 before the first blood culture and ending at their death on day 81. This case was previously included (case no. 10) in an outbreak report^11^. **C** Clinical isolates included in this study by hospital site, day of isolation, and CLSI (Clinical and Laboratory Standards Institute) MIC (minimum inhibitory concentration) values against anidulafungin as reported by NHS Microbiology laboratories.

We identified *C. auris* isolates from two patients with candidaemia refractory to echinocandin therapy treated in the intensive care units of St George’s (isolates StG1-5, Fig. 1A), and King’s College Hospitals (isolates K1-2, Fig. 1B) in London, UK. The first patient suffered persistent candidaemia for three successive days on treatment, whilst the second patient experienced an initial candidaemia, followed by breakthrough infection on echinocandin treatment after 33 days. Antifungal susceptibility testing performed by the UK Mycology Reference Lab in Bristol suggested that these fungal isolates were not echinocandin resistant (Fig. 1C) based on the tentative CLSI breakpoint ( ≥ 4 μg/mL) for anidulafungin in *C. auris*^60^. However, anidulafungin monotherapy failed to clear the candidaemia, and both patients required a switch to amphotericin B-based combination treatment and subsequently died, though neither death was directly attributed to candidaemia. Therefore, we sequenced the genomes of these *C. auris* isolates to explore the basis of their lack of response to echinocandin therapy.

**Fig. 2:**
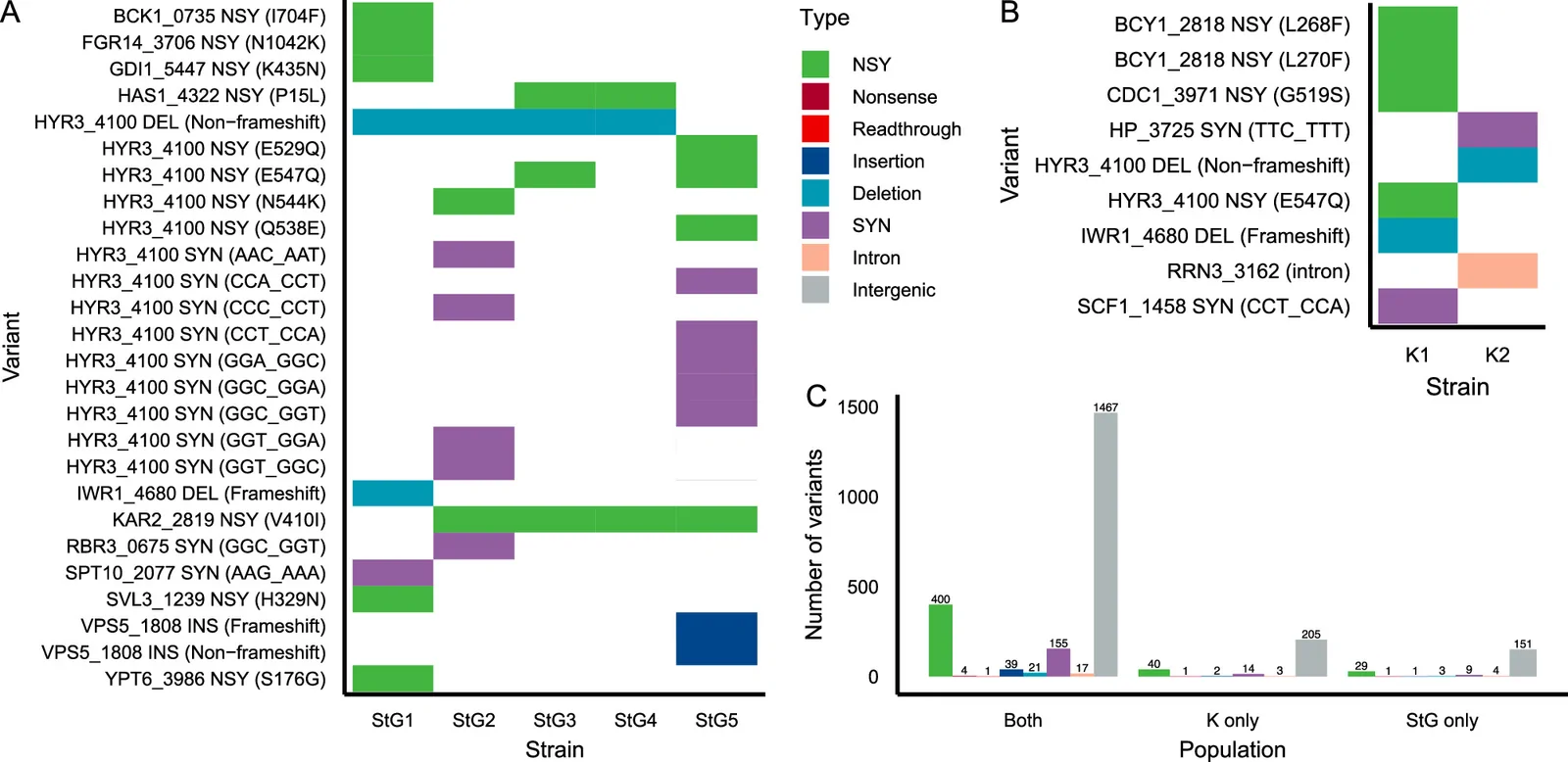
Common sequence variants identified in the genomes of the clinical series. **A** Genomic variants between clinical isolates in the StG1-5 series, excluding intergenic variants. **B** Genomic variants between clinical isolates in the K1-2 isolate series, excluding intergenic variants. **C** Counts of all genomic variants present across both clinical series. StG, St George’s Hospital case series. K, King’s College Hospital case series. Gene names are given in Supplementary Table S1. NSY non-synonymous, SYN synonymous, NON nonsense, INS insertion, DEL deletion.

To understand the genetic mechanisms underpinning echinocandin therapy failure in these two patients, we sequenced the genomes of their *C. auris* isolates. Whole genome sequencing revealed that each of the isolates belonged to clade I (Fig. S1) and, surprisingly, no non-synonymous mutations were present in *FKS1/2* genes. Therefore, we sought to understand additional pathogen-related mechanisms by which these infections were refractory to treatment. We identified 2567 single-nucleotide polymorphisms (SNPs) compared to the B8441 clade I reference genome, including 2104 in both StG and K series, of which 198 were present only in StG isolates, and 264 only in K isolates. Aside from SNPs in intergenic regions (71.0%), there were a total of 469 non-synonymous variants, 6 nonsense variants, and 66 indels in coding regions (Fig. 2A, B). The greatest variation across clinical isolate series was observed in *HYR3* (B9J08_004100: one deletion, four non-synonymous variants, and nine synonymous variants), which is predicted to encode a GPI-anchored cell wall protein^61^ (Fig. 2A, B). We also identified mutations in genes associated with cell wall stress response (*BCK1*), cell wall synthesis (*KAR2*, *RBR3*, *SCF1*), secretory pathways and intracellular trafficking (*GDI1*, *SVL3*, *VPS5*, *YPT6*), RNA synthesis (*HAS1*, *IWR1*), and histone acetylation (*SPT10*), suggesting genetic adaptations involving cell wall remodelling in response to drug pressure (Table S1). SNPs resulting in non-synonymous mutations were identified in genes related to azole resistance: *ERG11* (K143R), *CDR1* (V704L), and *TAC1b* (A640V) in isolates StG1-5; and *ERG11* (Y132F), *CDR1* (E709D), and *TAC1b* (A583S) in isolates K1-2. No non-synonymous mutations were observed in *MDR1-2*, *MRR1a-b*, *CAS5*, or *FUR1*.

We examined the genomes of the StG and K isolates (using thresholds of >1.4 and <0.6 normalised depth of coverage, Fig. S2A, B) to identify gene or chromosome copy number variation (CNV) associated with echinocandin refractory infection. Though we did not identify aneuploidy, small regions showing increased per-gene CNV included loci such as *NTO1* (part of putative histone acetyltransferase machinery) and *FGR14* (homologue of a retroviral endonuclease-reverse transcriptase). Areas of low copy number include multiple genes (e.g., *CDC13*, *ECM21*, *ECM42*, *HEM14*, *IWR1*, *MRF1*, *MRP49*, *PRD1*, *RCO1*, *TMA17*). To our knowledge, none of these CNV-affected genes have been associated with echinocandin resistance (Table S2).

### *C. auris* isolates display echinocandin heteroresistance and rapidly develop further resistance in vitro

**Fig. 3:**
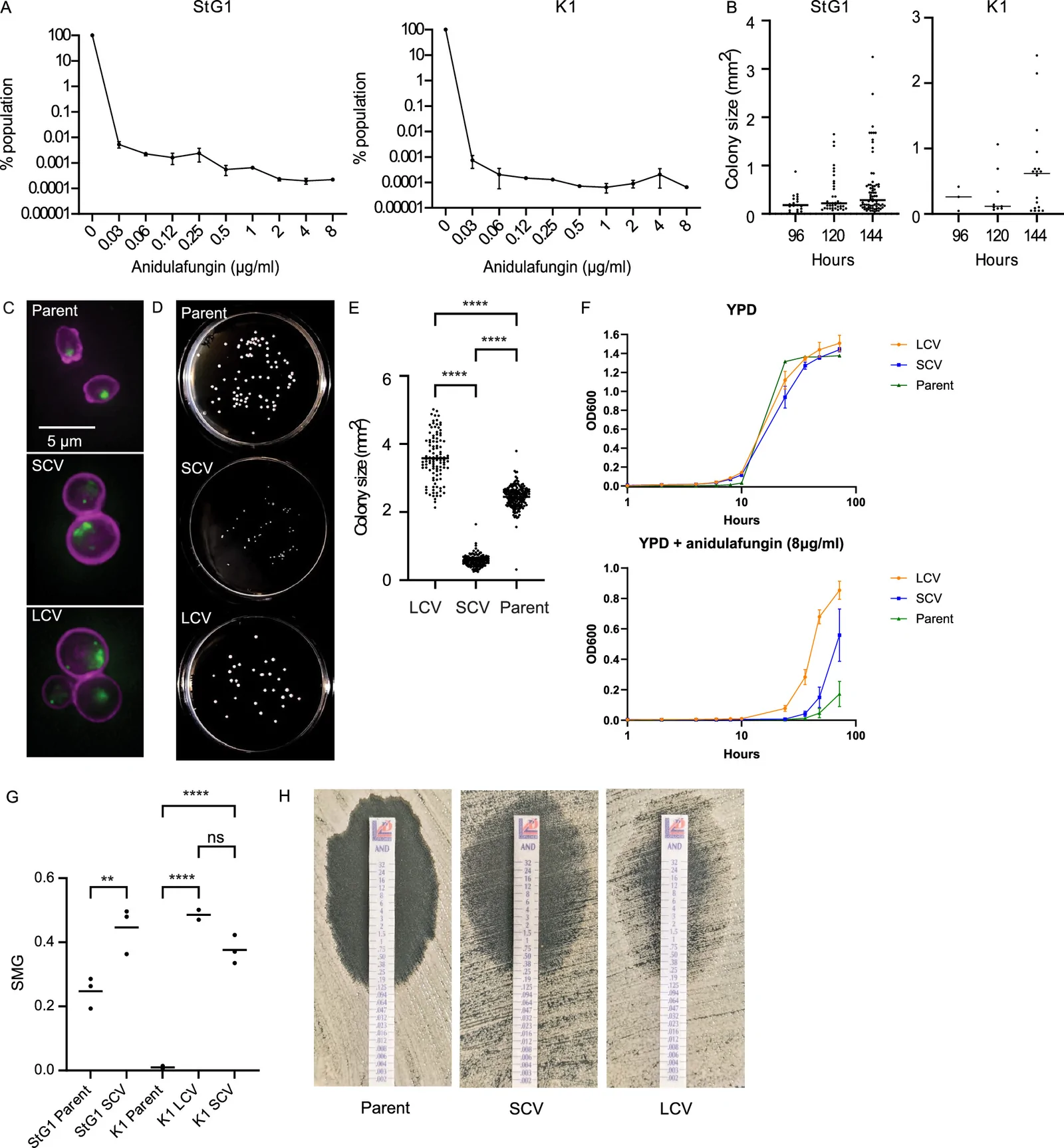
*C. auris* isolates display heteroresistance with sub-populations of cells displaying contrasting colonial morphotypes. **A** Population analysis profiling (PAP) of StG1 and K1 clinical isolates demonstrates anidulafungin heteroresistance. Points represent the mean of 3 biological replicates. Error bars represent the standard error of the mean (SEM). **B** Emergence of small and large morphotypes after protracted growth under drug pressure. StG1 and K1 isolates were plated onto YPD (yeast extract, peptone, dextrose) containing 8μg/mL anidulafungin, and colony sizes were measured using ImageJ after 96, 120, and 144h incubation (biological triplicates). **C** Fluorescence microscopy of individual cells, their cell walls stained with CFW (calcofluor white), and their DNA content with SYBR green. **D** Colony morphotypes of StG1 are stable following re-plating. StG1 large colony variants (LCVs) and small colony variants (SCVs) were taken from a YPD +anidulafungin 8 μg/mL plate, and the StG1 parental isolate was taken from a YPD plate. These cells were replated onto YPD at 10^2^ CFU and imaged after 48 h at 30 °C. **E** Colony sizes were then measured on these plates (**F**) by ImageJ. Plots represent the distribution of all colony sizes from 3 biological replicates for each cell type. One-way analysis of variance (ANOVA): *, *p* < 0.05; **, *p* ≤ 0.01; ***, *p* ≤ 0.001; ****, *p* ≤ 0.0001. **F** Growth (OD_600_) of the StG1 parent, LCV, and SCV colonies in YPD containing 0 or 8 μg/ml anidulafungin. Points represent the mean of 3 biological replicates, error bars represent SEM. **G** Supra-MIC growth for StG1 SCVs *vs* parent isolate. Each point represents the mean of technical triplicate results. One-way ANOVA, *p*-values as above. **H** Etest strips reveal the zones of inhibition for the parental isolate StG1, LCVs, and SCVs. StG, St George’s Hospital case series. K King’s College Hospital case series, MIC minimum inhibitory concentration.

Based on CLSI breakpoints, both StG and K isolates were echinocandin susceptible and yet obtained from clinically refractory infections. Therefore, we tested whether initial StG1 and K1 isolates displayed anidulafungin heteroresistance using population analysis profiling (PAP, Fig. 3A). Heteroresistance was indicated by the growth of small sub-populations of cells ( < 0.01%) on YPD-agar at anidulafungin concentrations 256-fold greater than those for the susceptible majority (Fig. 3A). After extended culture (144 h), two distinct colonial morphologies emerged that were most pronounced on YPD-agar containing 8 μg/ml anidulafungin (Fig. 3B): slower growing “small” colony variants (SCVs), and faster growing “large” colony variants (LCVs).

We compared the phenotypes of SCVs and LCVs: fluorescence microscopy revealed that, in the presence of anidulafungin, both SCVs and LCVs contained moderately larger cells than the parental isolate alongside aggregation and DNA content increase on SYBR green staining (Fig. 3C). The SCV and LCV morphotypes persisted when they were re-plated onto drug-free plates: re-plated SCVs (mean colony size 0.58 mm^3^) formed significantly smaller colonies (*p* < 0.0001) than LCVs (mean colony size 3.58 mm^3^) and parent isolates (mean size 2.45 mm^3^) following 48 h growth at 37 °C (Fig. 3D, E). SCVs and LCVs grew at similar rates to parental isolates in drug-free liquid YPD. However, in YPD containing 8 μg/mL anidulafungin, SCV cells grew significantly faster than parental cells, and LCVs even faster than SCV cells (Fig. 3F). Furthermore, both SCVs and LCVs were resistant to anidulafungin according to updated EUCAST criteria for *C. auris* (sensitive, S: ≤ 0.25 μg/mL; resistant, R: >0.25 μg/mL^27,28^): MIC against anidulafungin was highest for StG1 and K1 LCVs (modal MIC 4-8 μg/ml, range 2-16 μg/ml); followed by SCVs (modal MIC 2 μg/mL, range 1-4 μg/mL) and parental isolates (modal MIC 1-2 μg/mL, range 1–4 μg/mL, Table S3). SCVs also exhibited significantly higher tolerance to anidulafungin compared to the parent isolate, measured using both supra-MIC growth (Fig. 3G) and confirmatory Etest inhibition strips (Fig. 3H). LCVs were resistant according to Etest (Fig. 3H). According to the updated EUCAST guidelines, parental isolates were also resistant.

Taken together, these findings indicate the presence of stable heterogeneous sub-populations within treatment-refractory *C. auris* clinical isolates, which are consistently isolated at high anidulafungin concentrations. These distinctive small and large colonial morphotypes appear to reflect a spectrum of drug-adapted sub-populations, as shown by increased growth rates in the presence of drug, higher tolerance, and anidulafungin MICs- compared to the parent (rather than a difference in actual cell size).

### *CRZ1* mutations in LCVs are associated with anidulafungin resistance

We explored the genomic foundations of the colony morphotypes with associated tolerance and resistance phenotypes by sequencing SCV (*n* = 18) and LCV (*n* = 17) colonies. Mutations potentially underlying the observed population heterogeneity were identified by comparing their genomes with those of parental controls: StG1, K1, and K2 grown on YPD, with additional SCV/LCV isolates sequenced directly on anidulafungin at 8 μg/ml (*n* = 3 per isolate). No *FKS1/2* mutations were observed in any of the SCV or LCV genomes, indicating that anidulafungin resistance had emerged via *FKS1/2*-independent mechanisms.

**Fig. 4:**
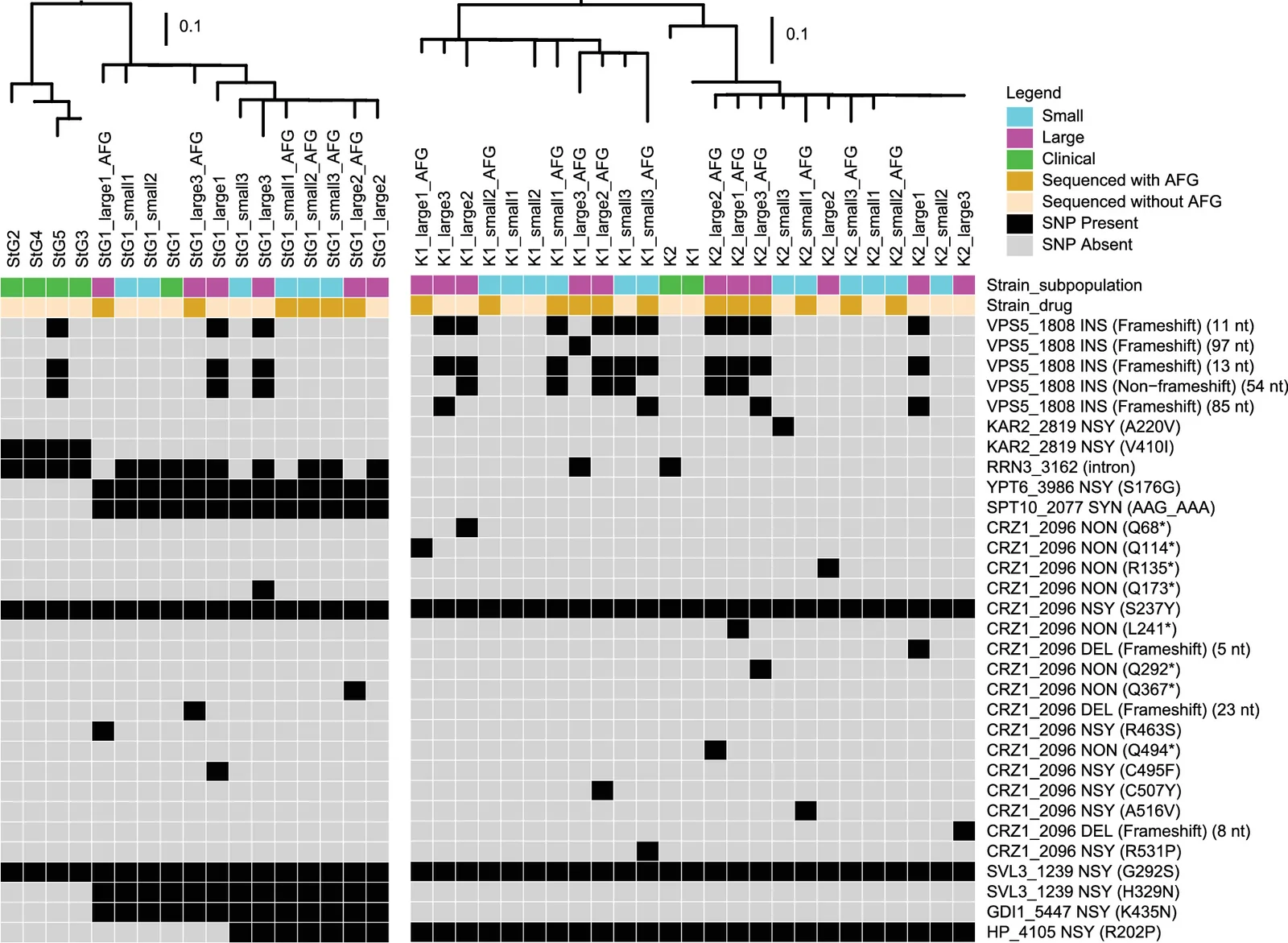
Significant within-gene variation in clinical isolates and their large and small morphotypes. Branch lengths indicate the mean number of nucleotide substitutions per site as per the scale bar. FastTree phylogenies for StG and K clinical series isolates are shown above. The colors in the legend (top right) and below the phylogenies indicate the group: clinical isolate versus large- or small-colony variant; genome sequenced following overnight growth on- or off-anidulafungin (8 μg/mL). Black boxes indicate the presence of the variant specified to the right of the figure: NSY: non-synonymous, SYN: synonymous, SNP: single nucleotide polymorphism, NON: nonsense, INS: insertion, DEL: deletion, AFG: anidulafungin. Differences between isolates K1 and K2 were driven by 237 intergenic variant differences (95 present only in isolate K1, 142 present only in isolate K2) in addition to those outlined in Fig. 2B. StG, St George’s Hospital case series. K, King’s College Hospital case series. Gene names are given in Supplementary Table S1.

To seek these *FKS1/2*-independent mechanisms, we examined sequence variants displaying a frequency difference of >25% between groups (e.g. SCV *vs* LCV, Fig. S3A) and compared the number of isolates in each group that displayed variants (Fig. S3B, C, Supplementary Note 1). Strikingly, variation in the *CRZ1* gene was significantly more common in LCVs compared to parent isolates (14/17 *vs* 0/7, 82.3% *vs* 0%, adjusted *p*-value = 0.032) and between LCVs and SCVs (2/18, 11.1%, adjusted *p*-value = 5.93 × 10^−5^). A total of 16 non-synonymous mutations were identified in *CRZ1*, excluding S237Y, which was present in all isolates (Fig. 4). The majority of *CRZ1* mutations were highly likely to alter function, including nonsense (*n* = 8) and frameshift deletions (*n* = 3), compared to non-synonymous mutations (*n* = 5). Thus, LCVs, which were anidulafungin resistant, carried the largest number of *CRZ1* mutations (Fig. 3F), consistent with the recent finding that *C. auris crz1* knockout mutants are anidulafungin resistant^62^.

The *CRZ1* calcineurin-responsive transcription factor gene was one of two genes in the calcineurin pathway that displayed highly significant variation in SCVs and LCVs. Another calcineurin-pathway related gene (*RCN2*, regulator of calcineurin) was significantly enriched for associated intergenic SNPs that flanked this gene in LCVs (88.2%) vs SCVs (27.8%, adjusted *p*-value 0.017). Furthermore, four frameshift mutations in *VPS5* (predicted vacuolar sorting protein) were identified in fourteen isolates, but these were not significantly enriched in SCVs or LCVs relative to the parental isolates. However, two non-synonymous variants were significantly enriched in daughters compared to clinical parents: *KAR2* V410I (*p* = 0.0077) and *HP_4105* R202P (*p* = 0.033). The change in Kar2 (a member of the Hsp70 chaperone family) could conceivably contribute to the tolerance profile of *C. auris* through stress response adaptations. However, the function of *HP_4105*, and hence the potential impact of the R202P mutation, remains obscure.

### Anidulafungin induces cell wall remodelling in both SCVs and LCVs

**Fig. 5:**
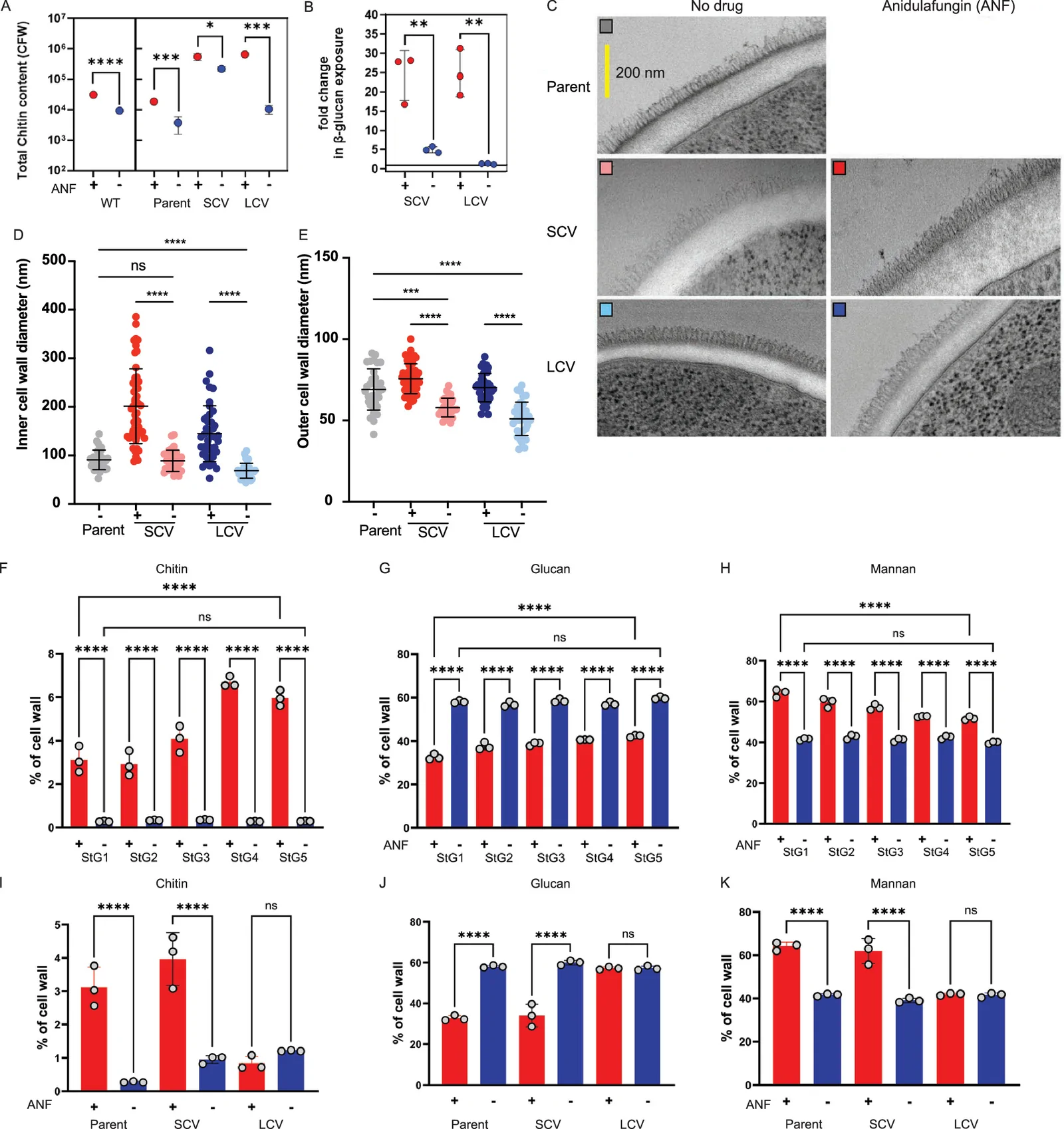
Impact of anidulafungin on chitin content and β−1,3-glucan exposure of cell walls of StG parents and large and small morphotypes. **A** The chitin content of the cell wall of StG parents and large and small morphotypes during growth in the presence (+, red) or absence (-, dark blue) of 8 μg/ml anidulafungin (ANF) was quantified by calcofluor white (CFW) staining and flow cytometry. Data represent *n* = 3 technical replicates, error bars represent mean +/- standard deviation (SD), statistics via two-sided unpaired t-test: ns, not significant; *, *p* < 0.05; **, *p* ≤ 0.01; ***, *p* ≤ 0.001; ****, *p* ≤ 0.0001. **B** The fold change in β−1,3-glucan exposure in large and small morphotypes, compared with parental isolates, in the presence (+, red) or absence (-, dark blue) of anidulafungin was quantified by dectin-1 staining and flow cytometry. Technical replicates were *n* = 3, statistics as per (**A**). **C** Transmission electron microscopy (TEM) reveals cell wall remodelling in response to anidulafungin. The diameters of the **D** inner and **E** outer cell wall layers were quantified from TEM images using ImageJ. Data represent means and standard deviations from 10 measurements per cell (*n* = 33, StG1 parent; *n* = 29, SCV -ANF; *n* = 53, SCV + ANF; *n* = 35, LCV -ANF; *n* = 43, LCV + ANF) and were analysed using two-sided Brown-Forsythe and Welch ANOVA, *p*-values as above. **F–K** High pressure ion chromatography (HPIC) with one-way ANOVA, *p*-values as above, *n* = 3 technical replicates, error bars representing mean +/- SD, measuring chitin (**F**), glucan (**G**), and mannan (**H**) contents of cell walls of the StG1-5 series following growth on yeast extract peptone dextrose (YPD) containing 0 or 8 μg/mL anidulafungin. Chitin (**I**), glucan (**J**), and mannan (**K**) contents of the cell walls of the parental isolate StG1 and its SCV and LCV isolates, and their corresponding changes in cell wall content inc. chitin, glucan, and mannan, after growth without or with (8μg/mL) anidulafungin. StG, St George’s Hospital case series. **K** King’s College Hospital case series. SCV, small colony variant. LCV, large colony variant.

In *C. albicans*, the inhibition of β−1,3-glucan synthesis by echinocandins induces compensatory increases in chitin synthesis and cell wall remodelling^63^, in part via Crz1 signalling^64^. In *C. auris*, caspofungin induces chitin synthase gene expression in a *CRZ1*-dependent fashion^62,65^. Therefore, we examined the impact of anidulafungin on the cell walls of LCVs and SCVs. Flow cytometry of Calcofluor-White (CFW) stained cells revealed that chitin levels increased in response to anidulafungin in both LCVs and SCVs (Fig. 5A), with associated increases in β−1,3-glucan exposure (Fig. 5B). Transmission electron microscopy (TEM) showed corresponding changes in cell wall architecture; exposure to anidulafungin led to significant thickening of the inner (chitin-rich) cell walls of both LCVs and SCVs (Fig. 5C–E). Anidulafungin also induced a slight increase in the outer mannan layer of the cell wall, but this was minor compared to the dramatic changes to the inner layer. High pressure ion chromatography (HPIC) of cell wall carbohydrates provided further evidence of anidulafungin-induced increases in chitin/mannan content with corresponding decreases in glucan both across both the StG clinical isolates (Fig. 5F–H) and in SCVs, but not in LCVs (Fig. 5I–K).

### Clinical and drug-tolerant/drug-resistant sub-populations of *C. auris* are characterised by alterations in ploidy

**Fig. 6:**
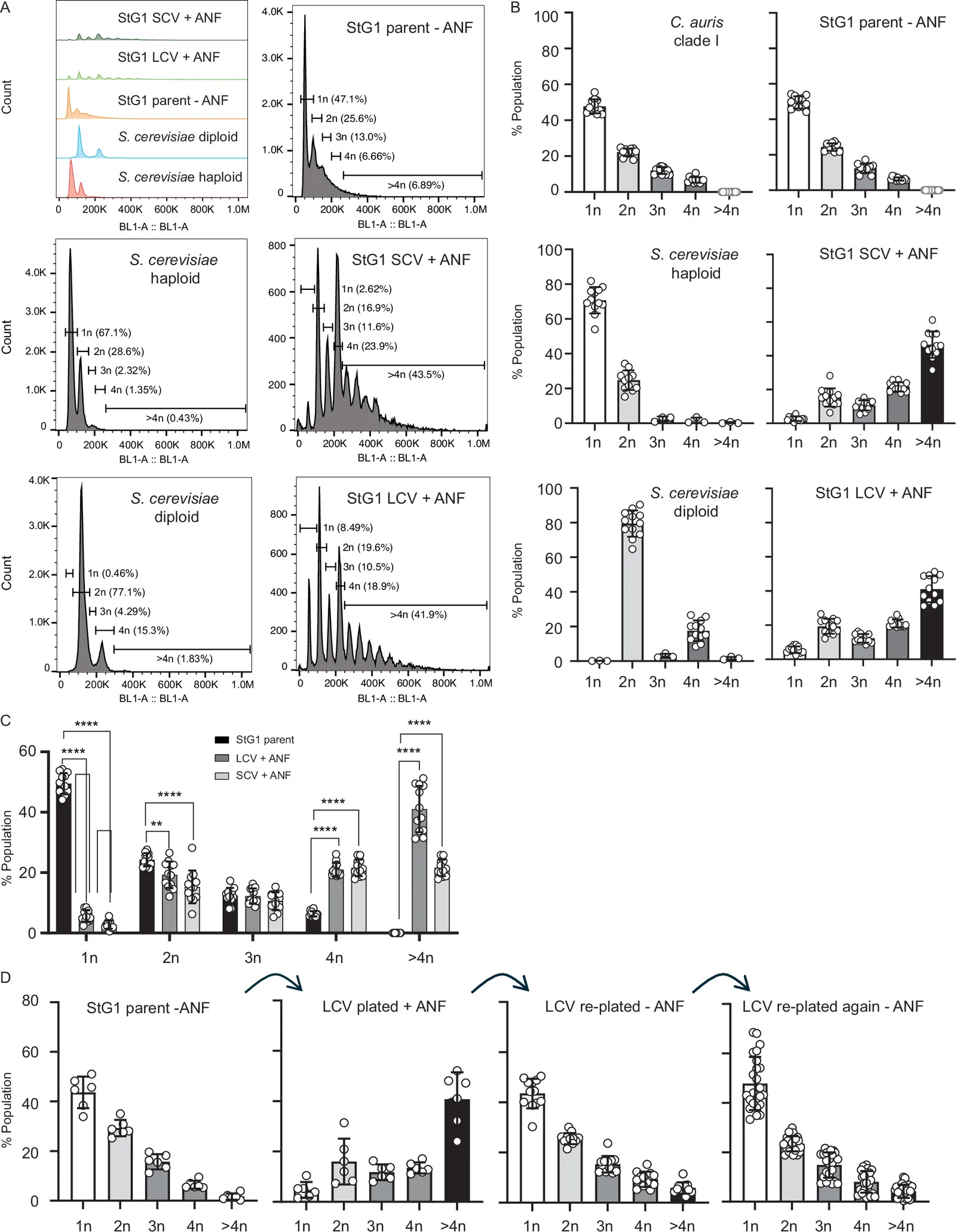
Exposure to anidulafungin increases the ploidy of *C. auris*. **A** The ploidy of *C. auris* cells in colonies taken directly from plates was measured by staining their DNA content with SYBR green and quantifying the fluorescence by flow cytometry. Haploid and diploid *S. cerevisiae* isolates and a *C. auris* clade I isolate were used as controls. Colonies for the *C. auris* clade I and *S. cerevisiae* controls were taken from yeast extract peptone dextrose (YPD) plates. Colonies for the *C. auris* StG1 parent and its small and large colony variants were harvested from YPD plates containing 8 μg/mL anidulafungin. Peak volumes were quantified using were quantified using FlowJo v.10 software. **B** The proportions of 1, 2n, 3n, 4n and >4n cells in each sample were calculated from the peak volumes obtained by flow cytometry in (**A**) with unpaired two-sided t-test comparison between groups (**C**): *p* < 0.05; **, *p* ≤ 0.01; ***, *p* ≤ 0.001; ****, *p* ≤ 0.0001. **D** The elevated ploidy of variants rapidly reverts to the parental ploidy following growth in the absence of drug. StG1 cells were plated onto YPD containing 0 or 8 μg/mL anidulafungin. After incubating for 5 days, parental colonies (*n* = 6) were taken from the drug-free plate, and LCV colonies (*n* = 6) from the drug-containing plate. The ploidies of cells in each of these colonies was quantified by SYBR green staining and flow cytometry. Cells from the same six LCVs were replated onto YPD without drug, grown for 1 day, and the ploidies of cells from two colonies quantified in duplicate (*n* = 12 in total). A second round of replating on YPD without drug was then performed using cells from these 12 colonies. After 1 day of growth, two colonies from each plate were selected and their ploidies quantified (*n* = 24 in total). All error bars represent mean +/- standard deviation (SD). StG, St George’s Hospital case series. K, King’s College Hospital case series. SCV, small colony variant. LCV, large colony variant.

Stress-induced changes in ploidy have been proposed to precede the emergence in drug resistance in *C. albicans*^66,67^, and tetraploidy has been described in *C. albicans* clinical isolates from human hosts^68–71^. Therefore, we compared the ploidies of *C. auris* StG1 and its SCV and LCV daughters, analysing cells taken directly from colonies on plates using flow cytometry: StG1 cells from YPD plates, and SCV and LCV cells from were taken directly from YPD plates containing 8 μg/ml anidulafungin. *Saccharomyces cerevisiae* haploid and diploid isolates were used as controls. The *S. cerevisiae* controls showed clean 1n-2n and 2n-4n ploidies, as expected for mixed populations containing cells pre- and post- S-phase (Fig. 6A, B). Although *C. auris* is purportedly haploid^72,73^, StG1 cells displayed heterogeneous ploidies ranging from 1n to >4n (Fig. 6A, B). Even higher ploidy ranges, with a higher proportion of cells with ploidies of >4n, were observed for the SCV and LCV morphotypes isolates from YPD+anidulafungin, differences which were statistically significant (Fig. 6C). Our bioinformatic analyses, which included allele frequency tallies (Fig. S4A), were consistent these increased ploidies, though identification of heterozygous sites was not sensitive to inter-isolate differences (Fig. S4B).

The heterogeneous ploidy of StG1 cells was unexpected (Fig. 6A, B). Therefore, to test whether other *C. auris* isolates display this phenotype, we examined isolates from clades I to V. Ten epidemiologically divergent isolates all displayed heterogeneous ploidies when grown on YPD agar (Fig. S4C) with strict single-cell gating strategies (Fig. S4D). Polyploidy was present in drug-unexposed isolates, suggesting that genomic instability and polyploidy may be features of *C. auris* underlying this species’ phenotypic heterogeneity and propensity towards drug tolerance/resistance, promoting treatment-refractory infection and emergence of secondary echinocandin resistance.

We reasoned that the elevated ploidy of variants, induced under drug pressure, might be lost following regrowth in the absence of drug. To test this, we grew colonies of the StG1 parent (*n* = 6) on drug-free plates and, in parallel, selected LCV colonies (*n* = 6) after 5 days of growth on YPD plates containing 8 μg/mL anidulafungin. We focused on LCVs rather than SCVs because this allowed us to examine the ploidy of individual variants (SCV colonies contain insufficient cells and had to be pooled to analyse ploidy). As before (Fig. 6A–C), cells in each of the six independent LCV colonies displayed elevated ploidies compared to the parental controls (Fig. 6D). We also replated cells from these six LCV colonies onto YPD lacking drug. After 1 day of growth without drug pressure, the ploidies of cells from two independent colonies derived from each six of the LCVs had reverted to the parental phenotype (*n* = 12 in total). Cells from these 12 colonies were then replated in the absence of drug, and two colonies from each plate were selected for analysis (*n*=24 in total). In all cases, the reversion to the parental phenotype was observed (Fig. 6D). We conclude that *C. auris* ploidy is transiently increased under drug pressure, and that this facilitates the emergence of mutations leading to drug tolerance (SCVs) or resistance (LCVs).

## Discussion

**Fig. 7:**
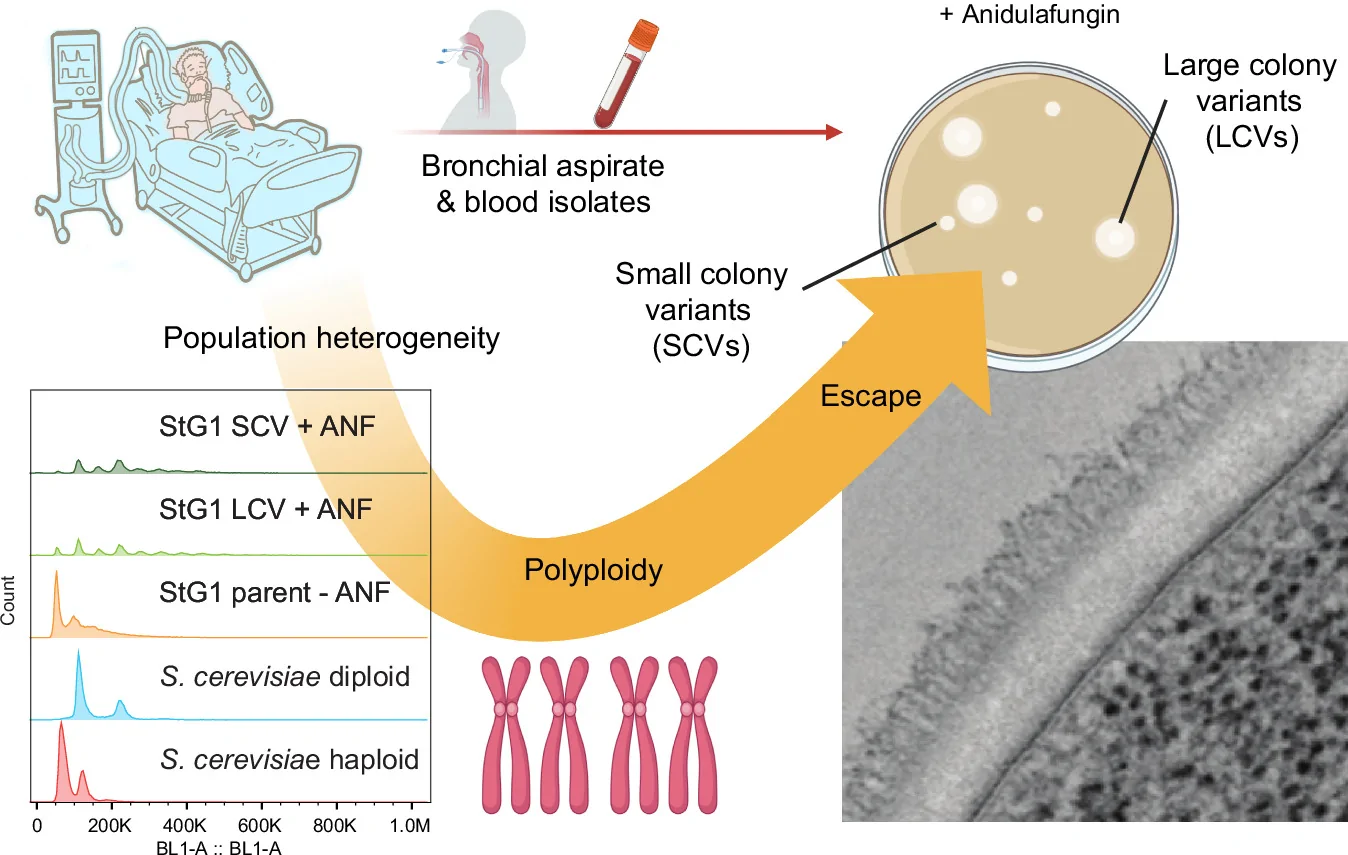
*FKS1/2*-independent mechanisms underlying the progressive emergence of resistance in echinocandin-refractory *Candida auris* infections. Illustration of ICU bed © the authors created in Procreate. Created in BioRender. Gifford, H. (2026) https://BioRender.com/vrusf9b.

In this study, we investigated serial invasive *C. auris* isolates from critically ill patients, which did not respond to anidulafungin despite the absence of *FKS* mutations. We have identified the presence of phenotypically heterogeneous sub-populations in *C. auris*, which display distinct colony morphotypes that show increased ploidy and enhanced adaptation to drug stress relative to the parent clinical isolate that reverts upon removal of drug (Fig. 7). The larger colony variant (LCV) grows faster in the presence of anidulafungin, has an elevated anidulafungin MIC, and carries significantly more *CRZ1* mutations than the small colony variant (SCV) and parental isolate. Whilst *C. albicans* and *C. glabrata crz1* mutants display echinocandin susceptibility^74–76^, a recent study has suggested a *C. auris crz1* null mutant is anidulafungin-resistant^62^. Consistent with this, *CRZ1* mutations observed in LCVs were nonsense/stop or deletion/frameshift mutations, which likely result in Crz1 disruption/dysfunction, and these were associated with increased anidulafungin resistance. *CRZ1* is likely to promote the fitness of *C. auris* under drug pressure, but *C. auris crz1* knockout isolates do not display attenuated virulence in a murine model of systemic candidiasis^62^.

Based on our findings, we propose that the following mechanisms contributed to the failure of anidulafungin therapy in the patients in our study. A small sub-population of drug-tolerant *C. auris* was able to survive high supra-MIC concentrations of anidulafungin (giving rise to SCVs). This subpopulation of cells was able to adapt to anidulafungin by increasing chitin synthesis and thickening their inner cell walls, which may have contributed to the drug-tolerant/intermediate-resistant phenotype of SCVs. We reason that the survival of these cells under drug pressure then enabled the emergence of genetic resistance, in part via *CRZ1* escape mutations, to yield the faster growing, anidulafungin-resistant LCVs. As standard variant calling methods exclude infrequent alleles, *CRZ1* escape mutations would be difficult to detect during the collection of clinical isolates, given the very low frequency of drug-resistant cells in heteroresistant isolates. The Crz1 transcription factor regulates cell wall genes in many fungi^76^, and therefore the loss of Crz1 functionality likely contributed to the observed blunting of the cell wall remodelling response of LCVs to anidulafungin.

The choice of an anidulafungin exposure concentration of 8 μg/mL is one dilution above the CLSI breakpoint of 4 μg/ml and reflects physiological concentrations identified in critically ill patients. Maximum plasma concentrations (C_max_) during critical illness range between 2.9-19.2 μg/mL between days 3-8 of anidulafungin therapy, with trough concentrations in the range of 1.0-4.7 μg/mL^77–82^. These concentrations support the selection of 8 μg/mL in this study as a clinically relevant exposure. The clinical isolates in this study were tested via CLSI methodology during the course of treatment by the National Mycology Reference Laboratory in Bristol to provide real-time results informing therapeutic decision-making (tentative breakpoint, ≥4 μg/mL^60^). We supplemented antifungal susceptibility testing by using the EUCAST methodology for this study, for which updated breakpoints were recently published, based on epidemiological data and visually inspected cutoffs (breakpoint, >0.25 μg/mL^28^). The impact on clinical decision-making and treatment failure through the use of different methodologies has not yet been fully addressed, but the reporting of both CLSI values (during clinical care) and EUCAST (for standardisation of isolates across research laboratories) enables a holistic understanding of the benefits and limitations of breakpoint determination methods.

Previous reports have suggested that echinocandin resistance in *C. auris* primarily arises via mutations in *FKS1/2* hot-spots in vivo^29–33^. We did not identify *FKS1/2* mutations in clinical isolates or sub-populations of SCVs/LCVs. Instead, we observed a predominance of *HYR3* variation within the clinically evolved case series. *HYR3* encodes a predicted GPI-anchored cell wall protein that is under selection in BSI-causing *C. auris* clades I, III, and IV^61^. Also, *HYR3* was highly upregulated in a murine catheter infection biofilm model in vivo^83^, and in mature biofilms with coincident echinocandin resistance^84^. Our findings reinforce the view that this locus is significant during human infection.

Variable ploidy is likely to be an important aspect of generating phenotypic heterogeneity in *C. auris*, and is perhaps an overlooked feature of genomic plasticity in this pathogen. Reports of polyploidy in *C. auris* are rare^73^, though diploid isolates have been reported from clades I and III, which are associated with higher virulence in a murine systemic infection model^85^. Polyploidy can accelerate genomic evolution and is viewed as a common and reversible fungal stress response that can increase the availability of beneficial mutations^44,45,68,86–88^ and even potentially increase virulence^66,89^. We provide evidence for drug-induced increases in ploidy in treatment-refractory *C. auris* isolates, strongly suggesting that changes in ploidy promote therapeutic escape by this important fungal pathogen^72,73^.

In light of our findings, we suggest updates to the three-stage model for the development of echinocandin resistance in *Candida* species^39,90^. We propose that the emergence of resistance begins with echinocandin tolerance driven by reversible physiological changes, for example, via protein kinase C, calcineurin-Crz1, HOG, and Hsp90 signalling^91^. Then, the exposure of sub-populations of physiologically relatively tolerant cells to sub-MIC echinocandin concentrations, especially in difficult-to-penetrate sites (e.g., catheter-related biofilms or intra-abdominal compartment, as was the case for our two critically ill patients), selects for the progressive emergence of resistance. Here, drug-induced increases in *C. auris* ploidy promote the accumulation of escape mutations. Additional drug pressure would then select for further mutations, for example in *FKS1* and/or *CRZ1*, that promote increased resistance and faster growth, thereby yielding the LCV phenotype. This updated model can account for the observed population heterogeneity of clinical isolates from patients that have undergone protracted drug treatments, where parental cells coexist with SCVs and LCVs displaying varying degrees of tolerance and resistance, and may represent parallel pathways towards overcoming antifungal pressure. This update may also inform the developing conceptualisation of bet-hedging in fungal pathogens^92^.

Our investigation of fungal population heterogeneity in patient isolates has revealed progressive pathways towards the emergence of echinocandin resistance in *C. auris* that may otherwise have been overlooked. The causes of treatment failure are complex, involving also drug delivery to the site of infection, as well as host immune dysfunction associated with critical illness. Nevertheless, defining the mechanisms underlying the emergence of drug tolerance and resistance in fungal pathogens, along with the temporal dynamics of their effects in vivo, may ultimately lead to improved patient outcomes. Therapeutic strategies could be tuned in real time, to minimise the development of drug tolerance or resistance in an individual patient^50,53^. Combination therapy is one such powerful option that could enhance efficacy, addresses intrinsic fungal population heterogeneity, and reduce the inherent risk of resistance selection by currently recommended echinocandin monotherapy^93,94^: for example, the ongoing Wellcome-Trust funded COMBAT *Candida* clinical trial (ISRCTN: 56596656) will compare micafungin alone to combination with micafungin and flucytosine for treatment of candidaemia in a setting of high *C. auris* prevalence in South Africa, incorporating resistance emergence as an endpoint.

In summary, we have uncovered pathogen-based mechanisms whereby *C. auris* sub-populations adapt to first-line therapy, contributing to persistence and evolution. The identification and association of *CRZ1* mutation with resistance provides a valuable candidate locus for future functional investigation, potentially serving as an early marker of resistance evolution. Future research should focus on validating *CRZ1* mutations by using structural biology, gene editing, and population genetics approaches. The clinical implications of our findings are that treatment-refractory *C. auris* infections require a more nuanced understanding of resistance beyond measurement of MICs, and that novel therapeutic strategies are needed to address the diverse adaptive mechanisms employed by *C. auris* under echinocandin pressure.

## Methods

### DNA extraction

Clinical isolates, which had been confirmed as *C. auris* using MALDI-TOF and tested for MIC by CLSI standards during the course of their treatment and not as part of this study, were stored at −80 °C in YPD containing 20% glycerol, and sub-cultured at 30 °C on YPD agar (Sigma Aldrich, UK). Yeasts were grown overnight in 10 ml YPD at 30 °C before centrifugation at 13,000 rpm, resuspension in 3 ml water, and transferring into a 2 ml tube. Cells were centrifuged at 13,000 rpm for 5 min. Prior to the supernatant being removed, the cells were resuspended in the residual liquid. Cells were snap-frozen by immersion in liquid nitrogen for 1 min. and then transferred into a 65 °C water bath for 3 min. Disruption buffer included 400 mg of glass beads, 200 μl of DNA extraction buffer (2% Triton X-100, 1% SDS, 100 mM NaCl, 1 mM EDTA, 10 mM Tris HCl (pH 8)), and 200 μl of phenol:chloroform:isoamyl alcohol (25:24:1), which was added to yeast samples, which were vortexed for four rounds of 20 s with 1 min. breaks on ice to cool samples. A sample of 200 μl 1x TE was added to the mixture before vortexing again for a few seconds and centrifugation at 13,000 rpm for 10 min. The aqueous layer was transferred into a fresh tube and mixed with 1 ml of 100% ethanol and centrifuged at 13,000 rpm for 5 min. The pellet was resuspended in 0.4 ml TE and 3 μl of 10 mg/ml RNAase A and incubated for 15 min at 37 °C. Samples of 10 μl of 4 mM ammonium acetate and 1 ml of 100% chilled ethanol were added. The mixture was centrifuged at 13,000 rpm for 10 min at 4 °C. The supernatant was discarded, and the pellet was resuspended in 100% chilled ethanol. The mixture was spun at 13,000 rpm at 4 °C for 10 min. The supernatant was discarded, and the pellet was dried in a heat block at 65 °C for 10 min. DNA was resuspended in 50 μl water. DNA concentration was checked using Nanodrop, and DNA quality was determined by gel electrophoresis.

### Sequencing and variant calling

Sequencing was performed by the University of Exeter Sequencing Service (ESS) on the NovaSeq 6000 with SP flow cell (Illumina, San Diego, USA). Quality control was performed with MultiQC v1.10.1^95^. Variant Calling was performed using the Genome Analysis Toolkit (GATK) v4.1.2.0^96^ with alignment of raw sequences to the B8441 v2 reference genome (GCA_002759435.2)^7^ and the mitochondrial genome (NC_053321.1)^97^ using BWA-MEM v0.7.17^98^. HaplotypeCaller was executed in GVCF mode with the haploid ploidy flag. Hard filters were used to remove spurious variants, including the filters quality by depth (QD) < 2.0, Fisher Strand (FS) > 60.0, and root mean square mapping quality (MQ) < 40.0.

### Population analysis profiling (PAP)

Overnight cultures of *C. auris* isolates were serially diluted 10-fold 6 times in sterile distilled water. 5 μl aliquots of each serial dilution were spotted in triplicate onto YPD agar plates infused with range of anidulafungin concentrations (0-8 μg/ml). Plates were incubated at 30 °C for 24-48 h and CFU counting performed to calculate CFU/ml able to grow on each drug concentration, which was then normalised to CFU/ml on drug free plate to calculate proportion of the population able to grow at each anidulafungin concentration. Due to noticeable heterogeneity in colony size at the maximum anidulafungin concentration (8 μg/ml), extended incubation of individual 8 μg/ml PAP plates inoculated with 10^7^ cells was performed. Plates were incubated at 30 °C for 7 days, and images taken after 48 h, 96 h, and 144 h. Colony sizes were measured manually using ImageJ at each timepoint. Briefly, images were converted to black and white, sharpened, and then the threshold was changed to remove parts of the plate with no colony growing. To assess stability of SCV/LCV phenotypes, replica plating of isolates was performed by preparing an inoculum of SCV/LCV in distilled water and then plating 102 cells on drug-free and 8μg/ml anidulafungin-containing YPD agar. Plates were incubated at 30 °C for 48 h, and colony sizes were calculated again using ImageJ as described previously.

### Growth curves

Microdilution plates were set up to contain 0, 0.25, or 8 μg/ml anidulafungin. Wells were inoculated with 1 × 10^5 ^log phase cells such that the final volume was 200 μL/ well. Plates were incubated at 30 °C for 50 h and constantly shaken at 100 rpm. OD_600_ was measured using a Spectrostar Nano every 10 min. for 50 h. Prior to OD_600_ reading, the plates were shaken at 600 rpm.

### Susceptibility and tolerance assays

Clinical isolates were tested at the point of isolation using CLSI methodology, confirmed at St. George’s and King’s College Hospitals, with confirmation by the National Reference Laboratory in Bristol. Additionally, the susceptibility of isolates to anidulafungin was determined per EUCAST guidelines with slight modifications. Briefly, 96-well plates were prepared to contain two-fold dilutions of anidulafungin (Gereon B1224), with the first and last wells containing no drug. Isolates were defrosted onto YPD agar and grown at 37 °C for 24 h. Five colonies were selected and resuspended in PBS and diluted such that 10^5^ cells were added to each well in a total volume of 200 μL/well, except the last. Susceptibility in RPMI 1640 media supplemented with 2% dextrose was tested at 37 °C. OD_450_ was read using a Tecan plate reader. The tentative clinical breakpoints were used to determine whether an isolate was susceptible or resistant. Plates were also read at 48 and 72 h to determine supra-MIC growth (SMG). SMG was determined as the average 72 h growth in the wells above MIC divided by the growth in the no-drug well. For Etest strips, isolates were grown for 48 h on YPD plates at 37 °C. Three to four colonies were taken and used to prepare 0.5 Mcfarland suspension. Sterile cotton swabs were used to streak each suspension across YPD plates in 3 separate directions to cover the whole plate in cells, and plates were allowed to dry. Sterile forceps were then used to place an Etest strip onto each plate, ensuring no air bubbles were present. Inoculated plates were incubated at 37 °C and were read and photos taken at 24 h and 48 h. Readings were made according to CDC guidelines for interpretation of Etest antifungal susceptibility testing results^99^.

### Phylogenomics

FastTree v2.1.11 with Jukes-Cantor modelling^100^ was used to construct phylogenetic trees based on multiple sequence FASTA alignments produced by ECATools^101^ using default parameters; midpoint rooted trees used 1,015 (StG clinical series plus laboratory evolved daughters) or 1,053 (K clinical series plus laboratory evolved daughters) phylogenetically informative sites. Additional sequences used to contextualise clinical samples within clades were obtained from the largest global genomic epidemiology study to date^23^ and the first five isolates were called and used for phylogenetic reconstruction, for which 192,032 sites were entirely covered in all.

### Statistical genomics and annotation

Copy number variation estimation and depth-of-coverage plots were calculated in comparison to normalised depth of coverage across the whole genome compared to the depth of coverage across either all positions for each gene locus or each 10 kb sliding window, from pileups created with Samtools mpileup^102^. Significance testing for variants/loci/functional annotations was performed using Fisher’s exact test (two-sided) with Benjamini-Hochberg (BH) testing for multiple correction with a cut-off of 0.05^103^. Significance testing for copy number variation was performed with Student’s T-test (two-sided), also with BH testing, but with a cut-off FDR of 0.005. Functional annotations and gene names were imported from prior analyses as described previously^104^. To identify the promoter site for Crz1 binding upstream of *FKS1*, we used Yeastract+^105^ to ascertain the presence of GNGGCKCA^106^ at contig PEKT02000002.1 position 1009439-1009439 (893-900 bases upstream from *FKS1* start) as the putative binding site for Crz1 (GAGGCCGCA). Contig edges were inferred using the B8441 v3 assembly, which demonstrates seven contigs^107^. To test for polyploidy, variants were re-called using GATK with the diploid flag to count heterozygous positions per 10 kb. Allele frequency counts were calculated from mpileups derived as above.

### Quantification of total chitin and β-glucan exposure

The chitin content in the cell walls of *C. auris* isolates and their segregants was compared using previously described methods^108^. Cells were grown in YPD at 30 °C for 5 h with or without 8 μg/ml anidulafungin, fixed with 50 mM thimerosal, and stained with 10 μg/mL Calcofluor-White (CFW) in the dark for 60 min. Stained cells were washed twice with PBS, and their fluorescence quantified using an Attune NxT flow cytometer. The plots represent three biological replicate experiments, in each of which 10,000 events were acquired. As a negative control, cells were treated as above without CFW. Median Fluorescence Intensities (MFI) were determined using FlowJo v.10 software. The exposure of β-glucan at the *C. auris* cell surface was quantified by flow cytometry as described previously^109^. Cells were fixed overnight with thimerosal and stained with Fc-Dectin-1 and anti-human IgG linked to Alexafluor 488 (Jackson ImmunoResearch, Ely, UK). The fluorescence of 10,000 cells per condition was assayed using an Attune NxT flow cytometer. Median Fluorescence Intensity (MFI) was quantified using FlowJo v.10 software, and fold changes in β−1,3-glucan exposure were calculated relative to the control.

### Transmission electron microscopy

Transmission electron microscopy (TEM) was performed as described previously^109^. Cells were subjected to high pressure freezing and freeze substitution^110^, and fixed and stained with 1% osmium. Ultrathin sections (70 nm) were prepared using lead citrate for contrast and imaged (JEOL 1400 JEM transmission electron microscope with Rio16 from Gatan, Ametek camera). Cell wall sections were imaged at a nominal magnification of x100k. For each condition, approx. 30 cells were imaged and 10 measurements of inner and outer cell wall diameter taken for each cell using the line tool in ImageJ^111^.

### Cell wall carbohydrate analysis

Determination of cell wall mannan, chitin, and b-glucan content was achieved by acid hydrolysing the polymers, and quantifying mannose, glucosamine, and glucose content, respectively, by high-performance anion-exchange ion chromatography with pulsed amperometric detection (HPIC) as previously described^112^.

### Ploidy testing

The ploidy of cells in *C. auris* colonies growing on YPD plates containing 8 μg/ml anidulafungin was assayed by flow cytometry using previously described procedures^72^. Using a toothpick, cells were harvested directly from individual colonies and fixed overnight in 70% ethanol. The next day, cells were harvested by centrifugation (8,000 rpm, 5 min.) and resuspended in 50 mM sodium citrate, pH 7.5 and incubated with RNase A (250 µg per 10^7^ cells) and proteinase K (1000 µg per 10^7^ cells) for 4 h at 37 °C. Cells were then washed in PBS, resuspended in 0.25% Triton-X 100 (Sigma-Aldrich) and stained with SYBR Green I (1:500; Sigma-Aldrich) overnight at 4 °C overnight. Before flow cytometry, samples were sonicated and washed with PBS. Flow cytometry was performed on an Attune NxT flow cytometer using an excitation wavelength of 488 nm. SYBR Green I fluorescence was detected with a 530/30 band pass filter and 50,000 events, gated for single cells, were recorded for each sample. The proportions of individual cells that displayed ploidies of 1n, 2n, 3n, 4n or >5n were quantified using FlowJo v.10 software, using isogenic haploid and diploid colonies of *S. cerevisiae* grown on YPD plates as controls: W303-1B a (*MAT****a****, ade2, his3, leu2, trp1, ura3*); W303-1B 2 M (*MAT****a****/MAT****α****, ade2/ade2, his3/his3, leu2/leu2, trp1/trp1, ura3/ura3*). The cells were also examined by fluorescence microscopy, staining them as described above with the addition of CFW (25 µg/ml) for 5 min. Cells were imaged using a DeltaVision Elite fluorescence microscope with a 60 x objective. Fluorescence excitation was generated by a Lumencor LED light source, and 10 μm Z-stacks of 50 images were captured by a pco.edge sCMOS camera. The 3D stacks were then deconvolved to remove out-of-focus light, and maximum intensity projections were used to create a 2D image using Image-J v1.54.

### Reporting summary

Further information on research design is available in the Nature Portfolio Reporting Summary linked to this article.

## Supplementary information

Supplementary Information

Reporting Summary

Transparent Peer Review file

## Source data

Source data file

## Data Availability

Raw reads generated in this study have been deposited in the SRA database under BioProject accession code PRJNA1373000. Further data generated in this study are provided in the Source Data file. Source data are provided with this paper.
