# Supplementary material for: FKS1/2-variant independent mechanisms underlying the emergence of resistance in echinocandin-refractory Candida auris infections
Source: Nat Commun. 2026 Oct 15;17:10406. doi: 10.1038/s41467-026-76954-x (PMC13672790; doi:10.1038/s41467-026-76954-x)
Supplement: Supplementary file 1 [file 41467_2026_76954_MOESM1_ESM.pdf]

FKS1/2-variant independent mechanisms underlying the emergence of resistance in echinocandin-refractory *Candida auris* infections: Supplementary information appendix

Hugh Gifford*[a+], Arnab Pradhan[a+], Ben Caswall[b+], Sarah Murphy[b+], Ian Leaves[a], Matt B. Edmondson[a], Tanmoy Chakraborty[a], Duncan Wilson[a], Surabhi K. Taori[c], Neil A. R. Gow[a], Rhys A. Farrer[a], Alistair J. P. Brown[a], Tihana Bicanic[a,b]

a: MRC Center for Medical Mycology at the University of Exeter, Geoffrey Pope Building, Stocker Road, Exeter, United Kingdom, EX4 4QD, UK

b: Institute of Infection & Immunity, St George's School of Health and Medical Sciences, City St George's University London, United Kingdom, SW17 0RE, UK

c: King's College Hospital, Denmark Hill, London, SE5 9RS, UK

+: These authors contributed equally.

*: Corresponding author - h.gifford@exeter.ac.uk

Supplementary note 1

In clinical isolates (StG1-5, K1-2), we also detected 178 synonymous variants, 42 insertions variants, 24 deletion variants, 24 intron-based variants, and 1 readthrough variant. We also identified three intergenic deletions (1-2 nt) across all clinical isolates located 664-1312 bases upstream of *FKS1*. Importantly, none of these mutations directly affected the putative Crz1 promoter binding site at 893-900 bases upstream but included GAA->G (664 bases upstream), CA->C (982 bases upstream) and TA->T (1,329 bases upstream). A further variable insertion (T->TAA-TAAAAAAAA, 1,527 bases upstream) was present in all clinical isolates except isolate StG2. These intergenic mutations may impact DNA conformation and interaction with promoter/binding sites, although this has specific interaction not been explored in *C. auris*. Only a single mitochondrial variant was present in the StG series, a 2-nucleotide insertion in isolates StG1-2 and StG3-4, with 11 separate small (1-3 base) intergenic substitutions/indels present across a series (isolates StG1-5:

n = 2, isolates K1-2: *n* = 2) or across both (*n* = 7).

Eight different nonsense mutations occurred exclusively in LCVs across the length of the gene, between amino acid position 68 and 494. Three different frameshift deletions between 5 and 23 bases also occurred exclusively in LCVs across the length of the gene, between amino acid positions 242-292, 367-463 and 516-531. Other mutations included R463S, C495F, and C507Y in LCVs and A516V and R531P in SCVs. *VPS5* also demonstrated significant variation in LCVs *vs* SCVs (58.8% *vs* 16.7%, adjusted *p*-value = 1.52×10^{-2}), with of up to three frameshift insertions of 11-97 bases in 9/17 LCVs, 1/7 clinical isolates and 2/18 SCVs.

We performed gene enrichment comparing those genes with significant variation between groups as defined above to the remaining genes using PFAM, KEGG and GO term annotation, GPI-anchor predictions, secreted protein and transmembrane domain predictions. After significance testing using Fisher's exact test using a Benjamini-Hochberg multiple testing cut-off of 0.05, We identified 76 PFAM, KEGG and GO terms driven by 24 loci that were enriched across the comparison between LCVs and SCVs (**Figure S5**). We did not identify any significantly enriched PFAM, KEGG and GO terms in the comparison between clinical and laboratory isolates. In addition to genes already discussed, this process highlighted variation in genes or associated intergenic regions for processes involving cell wall and membrane (*APS2*, *GDA1*, *TAZ1*), transcription and RNA processing (*IFH1*, *RAI1*, *STS1*, *UPT7*), DNA replication stress (*CAS1*, *LIA1*) and cellular morphology and cytoskeletal modelling (*OFD1*, *LOS1*, *PES1*, *CRN1*), adding to the candidate list for potential drivers of phenotypic variation in the comparison of LCVs and SCVs (**Table S4**).

Next, we compared copy number variation (CNV), using mean depth of coverage per gene locus across each genome in parent *vs* LCVs *vs* SCVs (**Figure S6A**). Intriguingly, the standard deviation for each locus was greater for LCVs and SCVs compared to clinical isolates (**Figure S6B**). There were ten loci with higher CNV in LCVs *vs* clinical isolates, and thirteen for SCV *vs* clinical isolates (**Figure S6C**). None of these areas of CNV reached above 1.98 normalised to genome-wide DOC, and all significantly

different DOC were higher in LCVs/SCVs compared to clinical isolates. Six loci had higher CNV in both, including cell wall components *CSA1* and *PGA6*, plus transcription factors *BCR1* and *HP_3966* (**Table S5**). Four loci had higher CNV in LCVs, including cell-wall related *IFF4*, and regulators of morphology *NUS1* and *WOR2*. Seven loci had higher CNV in SCVs, including mitochondrial *COR1*, histone acetyltransferase *NTO1* and metabolic genes *ACS1*, *RIB7*, and *SPE4*. These variable loci provide candidates where CNV may play a role in tolerance and resistance, though this CNV does not appear to entirely explain the LCV/SCV phenotypic variation.

With regards to the elevated copy number across three orthologues of the Zorro3 retrotransposon encoding *FGR14* in clinical isolates, and an area of increased copy number in chromosome 1 featuring histone modifier *NTO1*: the *FGR14*-related CNV has been described[1] and even associated with multidrug resistance[2], but the presence of repetitive elements could yet reflect assembly errors. Other areas of CNV implied the loss of genes involved in transcription (*ARX1*, *IWR1*, *MRP49*), DNA maintenance (*CDC13*, *MRF1*, *PRD1*, *TMA17*), and histone deacetylation (*RCO1*), raising the question as to whether these areas of CNV represent gene losses that could contribute to the echinocandin tolerance/resistance.

We also examined the influence of sequencing of isolates grown directly from overnight cultures with anidulafungin by testing differences between mean normalised depth of coverage (DOC) for each locus by each group (student's T-test with Benjamini-Hochberg correction and false discovery rate (FDR) <0.005). There were no loci with significant differences in CNV between isolates sequenced directly on or off anidulafungin, consistent with the assumption that the genotype/phenotype of variants was preserved through generations.

The clear predominance of different *CRZ1* mutations in LCVs indicates a role for the calcium-calcineurin stress signaling pathway in resistance. Intergenic-associated mutations for *RCN2* were also present in LCVs. This pathway is implicated in echinocandin resistance mechanism development, given that the Crz1 transcription factor

plays a role in cell wall remodeling e.g. *via* modulating *FKS* and *CHS* gene expression in *S. cerevisiae*[3,4].

Our findings of highly variable copy number across loci in LCVs/SCVs could be linked to the polyploid state, potentially including significant genome instability across isolates. The loci involved led us to hypothesise for roles in transcription factor and biofilm regulator *BCR1*, and cell wall genes components *CSA1* and *PGA6*, in resistance/tolerance. Furthermore, the morphology regulators *WOR2* and *NUS1*, which are implicated in *C. albicans* white-opaque switching and filamentation respectively[5,6], had higher CNV in LCVs. The higher CNV of metabolic, mitochondrial and histone acetyltransferase-related genes in SCVs also lends weight to the possibility that polyploidy-driven genome instability may confer drug resistance/tolerance through multiple mechanisms.

We used a range of techniques to explore the dynamic cell wall changes, including TEM and HPIC. We found that LCVs/SCVs had cell walls with an increased inner (glucan-chitin rich) cell wall diameter, while cells from LCVs exhibited a high glucan content, implying escape from glucan synthase inhibition in the face of anidulafungin drug pressure. Cell wall remodeling and chitin synthesis in *C. albicans* is regulated in part by *CRZ1* signaling[7]. Echinocandin treatment stimulated increases in cell wall chitin content *via* cell wall remodeling in *C. albicans*, analogous to our findings[8], with an elevated exposure of their pro-inflammatory β-glucan pathogen-associated molecular patterns (PAMPs)[9]. Echinocandin tolerance has long been associated with increased chitin content as a response to enable cell wall strengthening[10]. A recent solid-state NMR analysis of *C. auris* after caspofungin exposure indicated increases in cell wall strength through rigidification of mannan and β-1,3-glucan sidechains, rather than through increased chitin content[11]. We find that increased glucan content of cells from LCVs suggests an alternative adaptation mediated by *CRZ1* polymorphisms.

We considered the hypothesis that SCVs were petite mutants. Petite colony morphotype mutants are defective in mitochondrial respiration and may not confer

echinocandin resistance but may prevent synergy of caspofungin with calcineurin inhibitors e.g. in *C. glabrata*[12]. SCVs have been described in *C. glabrata* in echinocandin microevolution experiments. These were associated with echinocandin resistance and preserved virulence, but with no apparent respiratory function deficiency[13]. We did not identify any mitochondrial genome mutations in our echinocandin resistant or tolerant sub-population, or any significant differences in intergenic variation between colony morphotypes. The petite phenotype is a respiration deficient variant demonstrating a small colony morphotype, that can be demonstrated through mitochondrial variants or genomic defects in oxidative phosphorylation[14] with reduced growth in YPD liquid or YPD-glycerol of *via* violet agar testing[15]. In *C. glabrata*, the petite phenotype does not confer resistance and has lower *in vivo* fitness[12] but may confer persistence in response to echinocandin exposure[16].

Several unexplored techniques to interrogate *C. auris* resistance/tolerance here include the transcriptome (to assess physiological difference) and epigenome (to understand chromatin changes and histone modifications). Investigating these avenues is supported by our data, which provide evidence of variation in transcriptional and chromatin/histone-related machinery. Epigenetic regulation of resistance in *C. auris* through chromatin modelling has not yet been fully evaluated, and this is an obvious next step in investigation following our findings. Roles for histone modification in *C. albicans* stress tolerance[17] and azole drug resistance[18] make it highly plausible such mechanisms could underlie resistance in *C. auris*.

Supplementary figures

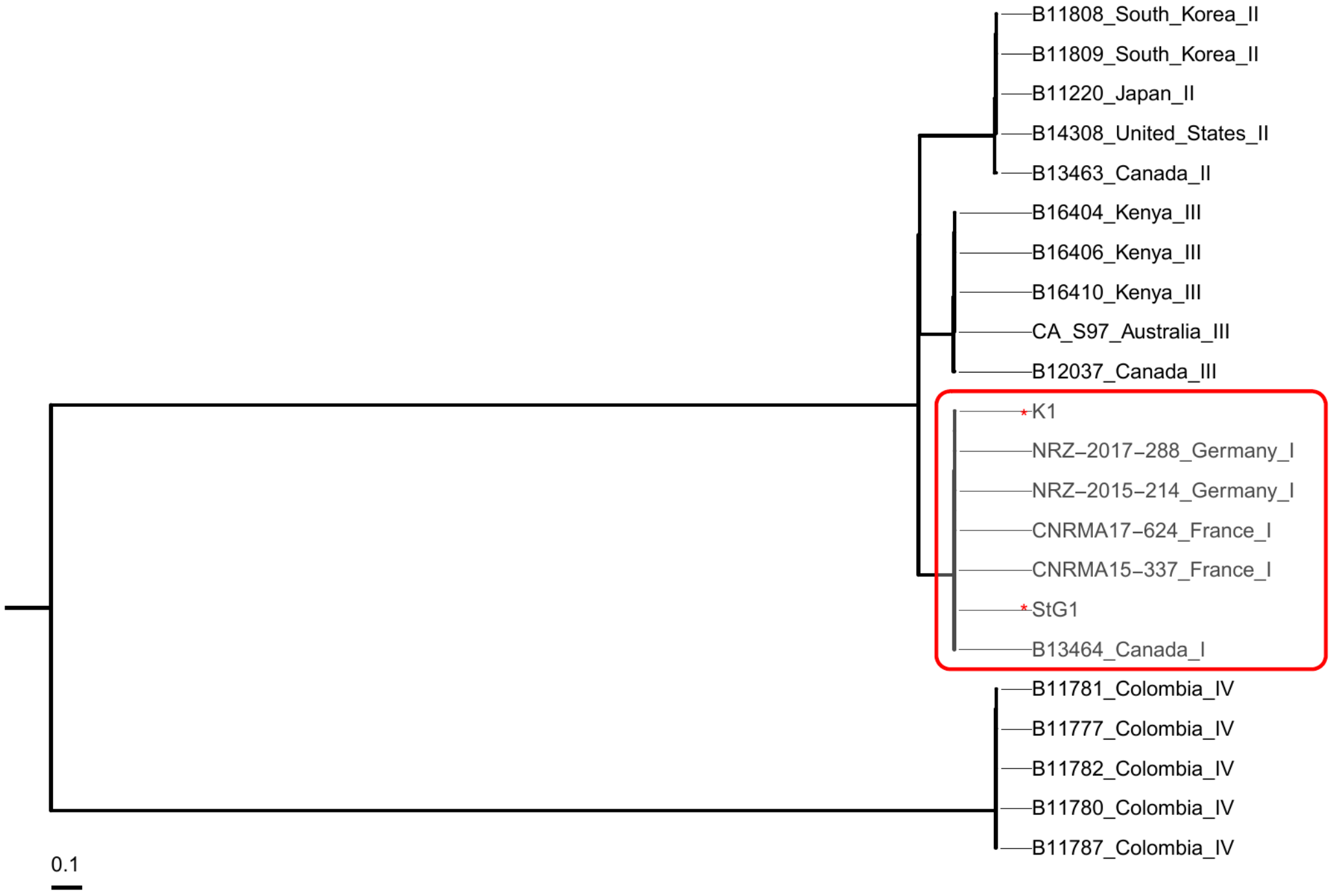

Figure S1: FastTree phylogeny demonstrating the placement of both parent clinical isolates (red asterisks) isolate StG1 (St George's Hospital) and isolate K1 (Kings College Hospital) and within clade I (red box). Isolates were randomly chosen as representatives of clades I-IV from a previous study[19]. Other isolates are designated by their isolate name, country of isolation, and known clade placement. Scale bar indicates number of substitutions per site.

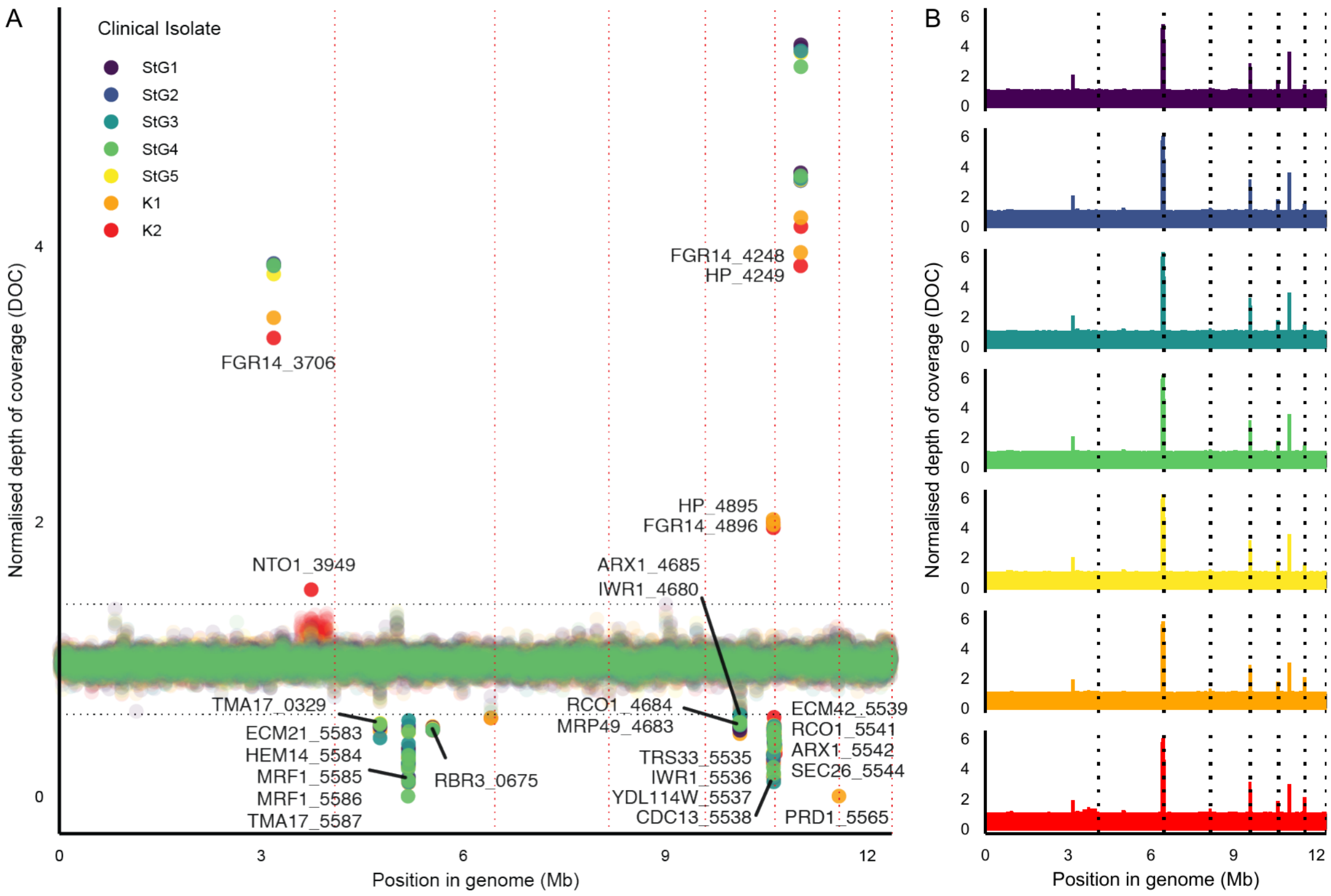

Figure S2: Clinical series copy number variation: **(A)** Copy number variation estimation by normalised depth of coverage per gene. Black horizontal line indicates cut-off (0.6/1.4), red vertical lines indicate contigs. **(B)** Normalised depth of coverage across the whole genome for each clinical isolate in 10 kb sliding windows. Red vertical lines indicate contigs. NSY: non-synonymous, SYN: synonymous.

149

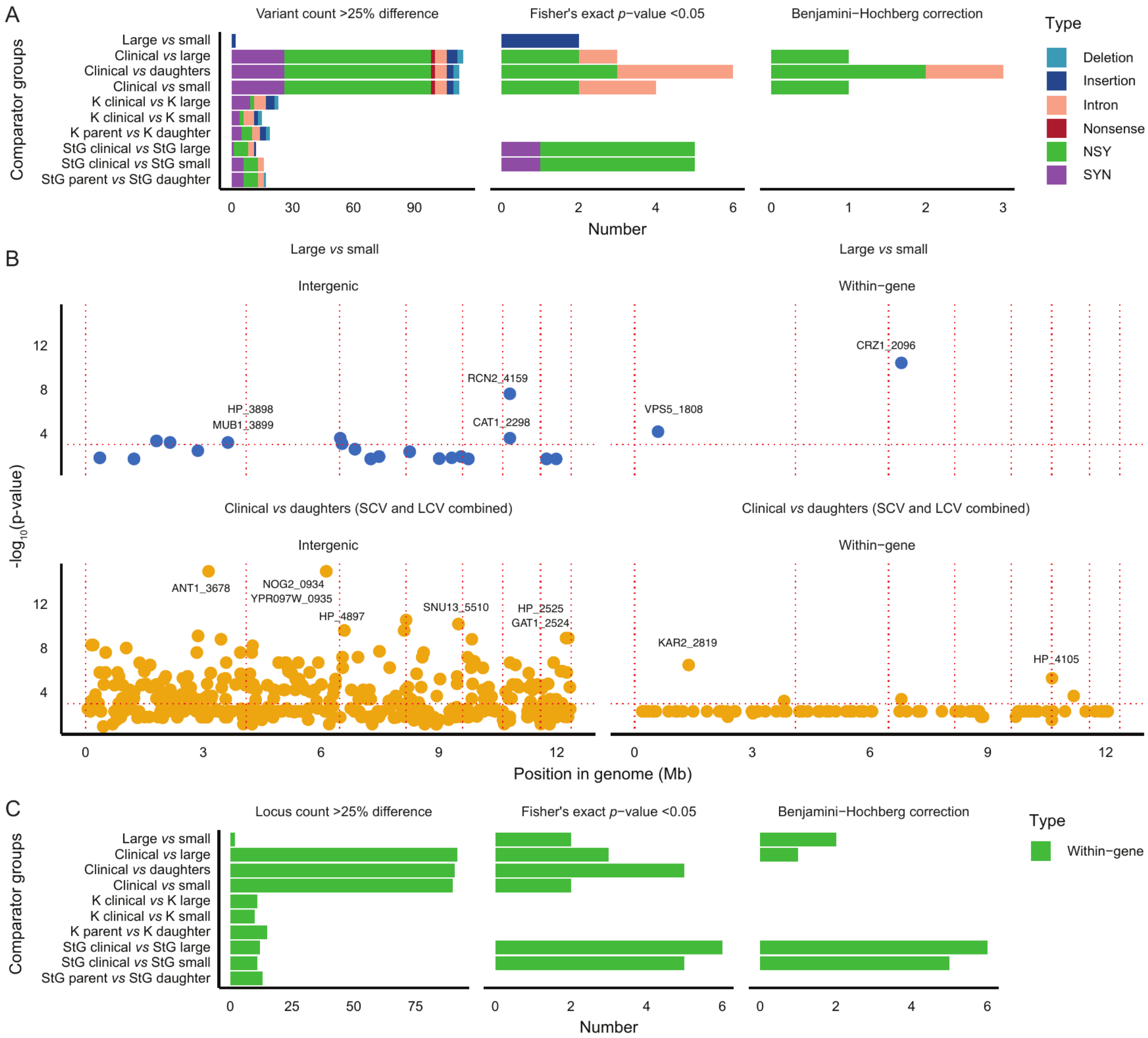

Figure S3: Genome-wide association: **(A)** Number of specific variants with >25% difference in proportion in between groups, tested for significance by Fisher's exact test with BH correction for within-gene variants. **(B)** Variable loci (genes with any variants) significant after BH correction plotted along genome position, with contigs marked in red. **(C)** Number of gene loci containing any variants with >25% difference in proportion, tested for significance as above. NSY: non-synonymous, SYN: synonymous.

150

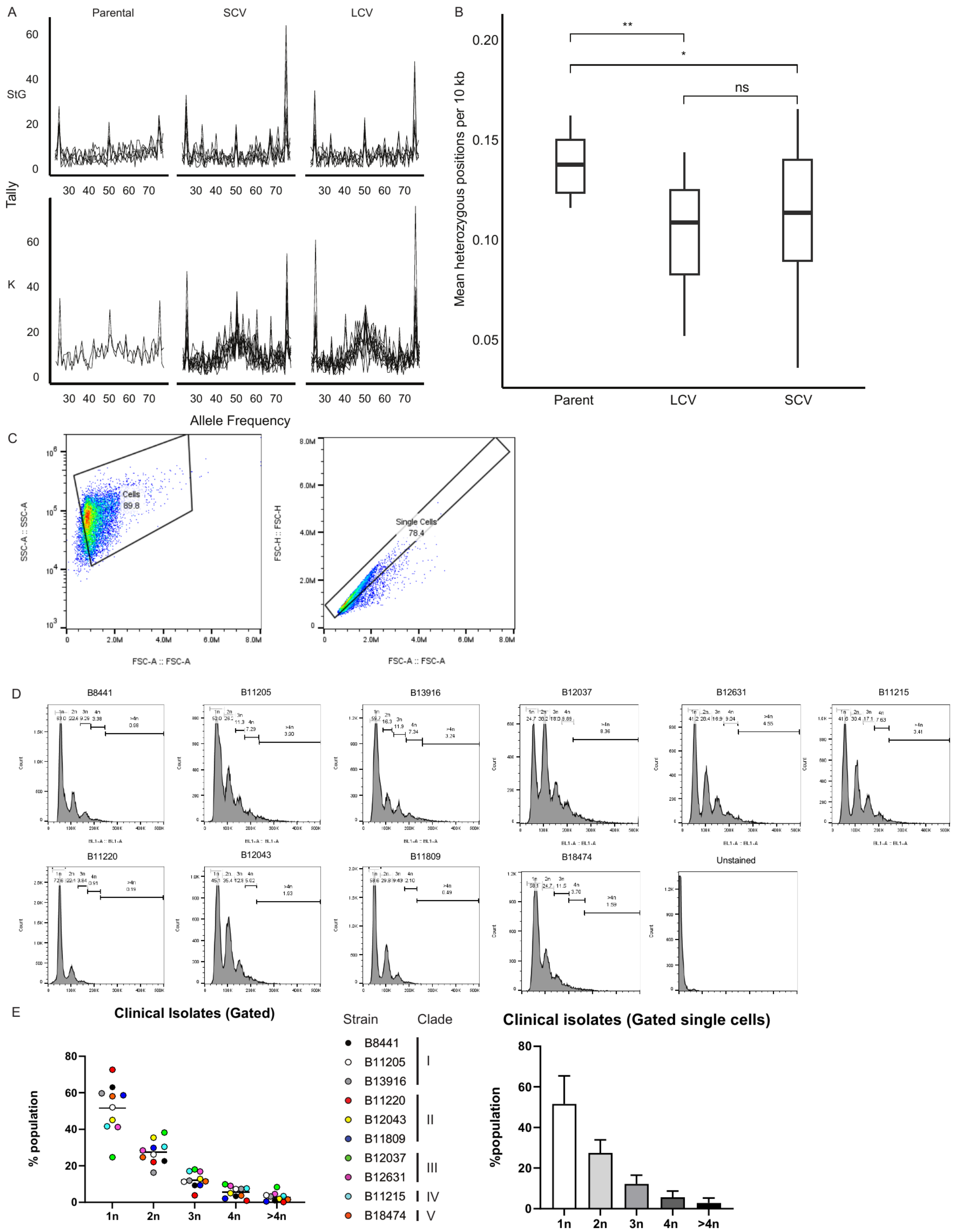

Figure S4: Bioinformatic and flow cytometric testing for polyploidy in *C. auris*. **(A)** Allele frequency tallies for each isolate, grouped by colony morphotype and clinical series. **(B)**

Numbers of heterozygous sites after variant calling with diploid flags. Boxplot indicates median, interquartile ranges as hinges, and whiskers extending to maximum value <1.5 times the interquartile range from the hinges. Significance levels indicate two-sided T-test, * = *p* <0.05, ** = *p* ≤0.01. **(C)** Single cell gating was performed by using the FSC-A vs FSC-H strategy. **(D-E)** Test isolates from epidemiologically derived clinical *C. auris* isolates, clades I-V[20], and frequencies of elevated ploidy.

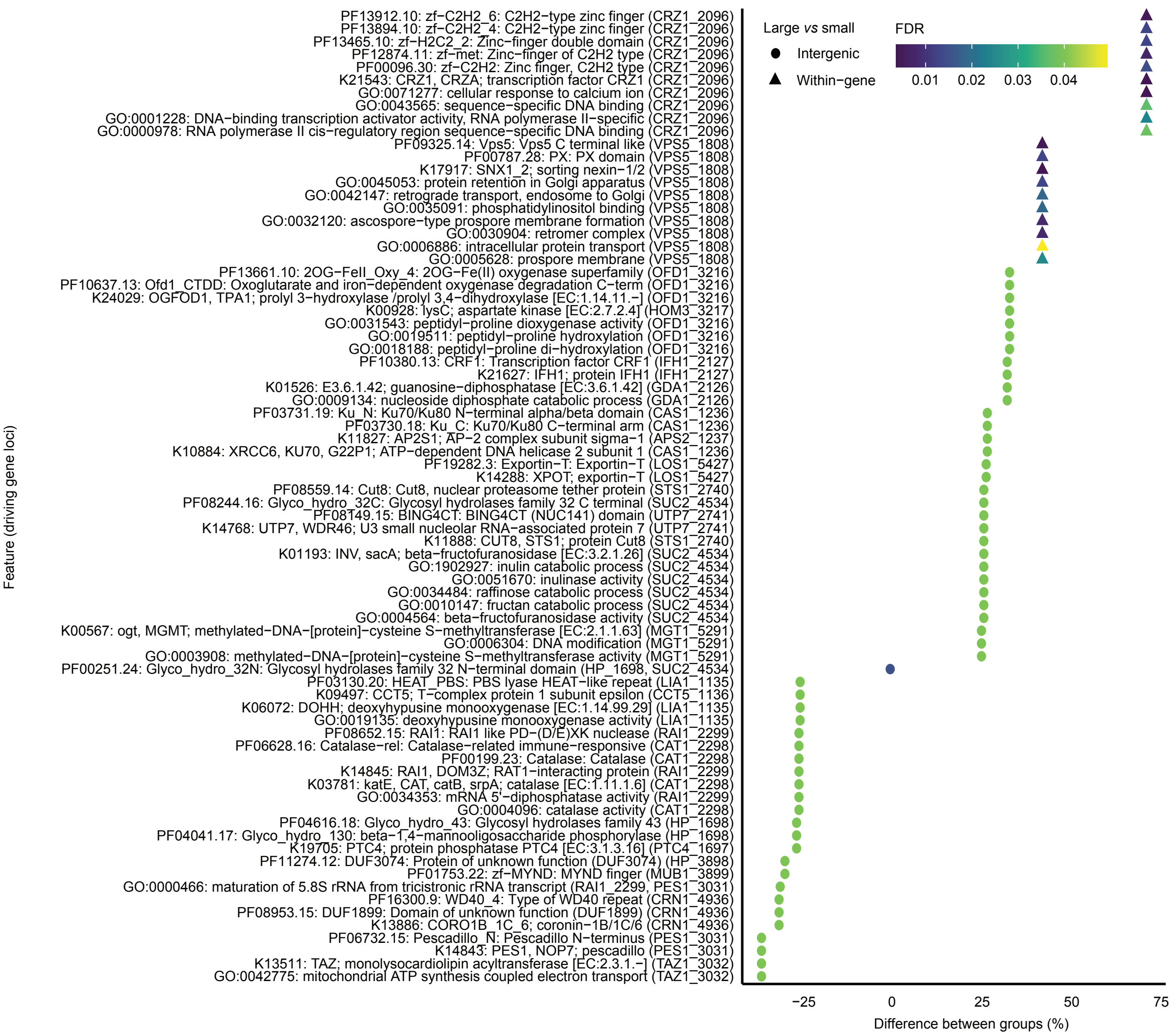

Figure S5: Loci annotated with GO terms, KEGG pathways and PFAM domains were tested for enrichment between LCVs and SCVs. Driving genes for each enriched term is indicated in brackets.

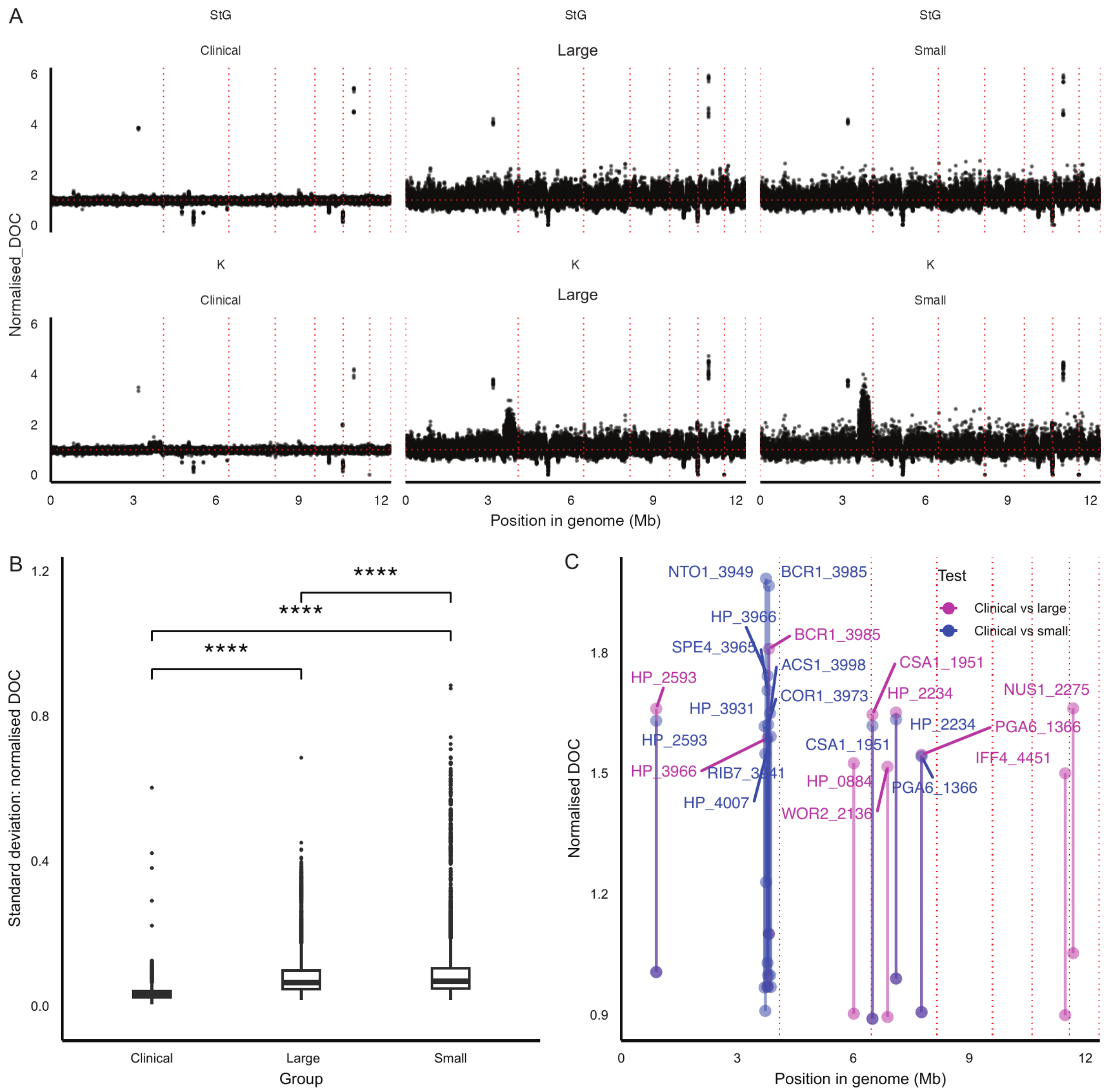

Figure S6: Copy number variation testing in morphotypic variant isolates: **(A)** Normalised depth of coverage (DOC) per gene locus for each isolate derived from StG and K clinical case series. **(B)** Standard deviation for each locus' normalised DOC (Wilcoxon test, significance level indicates p<0.0001). **(C)** Areas of CNV that were significantly different (>0.6 difference in normalised DOC, student's T-test with Benjamini-Hochberg correction

and false discovery rate (FDR) <0.005) on comparison between large colony variants (LCVs) and small colony variants (SCVs) with clinical isolates.

Supplementary tables

Gene	Stands for	Function in *C. auris*	Function in other human fungal pathogens
BCK1_0735	Bypass of C Kinase[21], member of Pkc1 cascade as mitogen-activated protein kinase kinase kinase (MAPKKK), cell wall stress stress response	Upregulated in response to caspofungin stress[22]	Deletion mutant in *C. albicans* is Caspofungin sensitive[23]
BCY1_2818	Bypass of cyclic AMP requirement[24] Protein kinase A regulatory subunit	Deletion associated with major loss of adhesion[25] and reduced virulence[26]	Deletion mutant in *C. albicans* has increased filamentation and white-opaque switching[27]
CDC1_3971	Cell division cycle protein[28]	-	Modification of the GPI anchor to facilitated glucan and cell wall protein binding across *Candida*[29]
FGR14_3706	Filamentous Growth Regulator[30] retroviral endonuclease-reverse transcriptase	Putative copy number variant[1]	Multiple putative functions involved in filamentation[30]
GDI1_5447	GDP-dissociation inhibitor[31]	-	Essential for secretory pathway and chitin deposition in *A. niger*[32]
HAS1_4322	Helicase Associated with Set1[33] involved in 18S rRNA biogenesis	-	Some evidence of variable expression in echinocandin resistant *C. albicans*[34]
HP_3725	Hypothetical protein	-	Orthologous to *C. albicans* translation initiation factor 33[35]
HYR3_4100	Hyphally regulated[36] GPI-anchored cell wall protein, also named Hil6[37]	Highly upregulated in murine catheter infection biofilm model *in vivo*[38]; upregulated in mature biofilms with concurrent echinocandin resistance[39] associated with efflux pump expression but also thought to be involved in extracellular matrix sequestration[40] and with signatures of selection in clades I, III and IV[41]	Mutations in areas of LOH in *C. albicans* serial clinical isolates with variable azole resistance[42]
IWR1_4680	Interacts With RNA polymerase II[43] putatively involved in nuclear protein import	-	-
KAR2_2819	KARyogamy 2 [@] Hsp70 family ATPase	-	Putative role in cell wall beta-glucan synthesis (GO:0070880) and ER translocation
RBR3_0675	Repressed by *RIM101*[44] cell wall gene	Mutations identified in clinical echinocandin resistance evolution[45], signatures of selection in clades I, III and IV[41]	Adhesin linked to glucan networks in *C. albicans*[46]
SCF1_1458	Surface colonisation factor1[25], also known as RBT1	Surface colonisation factor1[25], required for adhesion and virulence and highly upregulated in murine catheter model *in vivo* biofilm[38]	Represented in network analysis of *C. albicans* gene expression network in caspofungin exposure[47]
RRN3_3162	Regulation of RNA polymerase I[48]	-	Understood to be upregulated in *C. albicans* on exposure to caspofungin[49], lost in fluconazole evolution experiment in *C. albicans*[50]
SPT10_2077	SuPpressor of Ty[51] Histone H3 acetylase	Upregulated in Amphotericin B resistant strains[52]	Putative target for antifungal therapy across *Candida* species[53]
SVL3_1239	Styryl dye Vacuolar Localization[54]; role in endocytosis	-	-
VPS5_1808	Vacuolar Protein Sorting 5[55]	-	-
YPT6_3986	Yeast Protein Two[55] endosome-to-golgi-mediating Rab family GTPase	-	Mutations can confer resistance to azole synergisers in several yeast species[56]

Table S1: Variable loci in clinically evolved isolates, with gene name details, studies relating to function in *C. auris*, and role of orthologues in related *Candida* species. Gene

names include final four number from B9J08_00 locus tags from the B8441 V2 reference strain.

Gene	Stands for	Function in *C. auris*	Function in other human fungal pathogens
ARX1_4685	Associated with Ribosomal eXport complex[57]	Putative DNA-binding protein	Putative DNA-binding protein
ARX1_5542	”	”	”
CDC13_5538	Cell Division Cycle[28]	-	Telomere binding and protecting across *Candida* sp.[58]
ECM21_5583	ExtraCellular Mutant[59]	-	Regulator of endocytosis, down-regulated in *C. albicans* in response to caspofungin[49], predicted to be involved in cell wall assembly with modelling of glucan synthesis gene expression networks and hypersensitivity to calcofluor white in mutant *S. cerevisiae*[60]
ECM42_5539	”	-	Putative role in ornithine acetyltransferase/arginine biosynthesis, upregulated in amino acid starvation[61]
FGR14_3706	Filamentous Growth Regulator[30] retroviral endonuclease-reverse transcriptase	Putative copy number variant encoded by Zorro3 retrotransposase[1], part of a core orthologous group associated with azole resistance suggested to play a role in muti-drug resistance via regulation of transcription/RNA life-cycle as an RNA-dependent DNA polymerase[2]	Multiple putative functions involved in filamentation[30]
FGR14_4248	”	”	”
FGR14_4896	”	”	”
HEM14_5584	HEMe biosynthesis[62]	-	Upregulated in transcriptome of azole-exposed *C. glabrate*, thought to play a role in increased Erg11 activity[63]
HP_4249	Hypothetical protein	Putative zinc-binding transcription factor, identified as being associated with amphotericin B and fluconazole resistance[2]	-
HP_4895	”	-	-
IWR1_4680	Interacts With RNA polymerase II[43] involved in protein import into nucleus	-	Nuclear import of RNA polymerase II in *S. cerevisiae*[64]
IWR1_5536	”	-	”
MRF1_5585	Mitochondrial peptide chain Release Factor[65]	-	Disruption leads to mitochondrial genome instability in *S. cerevisiae*[65]
MRF1_5586	”	-	”
MRP49_4683	Mitochondrial ribosome protein[66]	-	Component of large ribosomal subunit[66]
NTO1_3949	NuA Three Orf[67]	-	Putative histone acetyltransferase (HAT) complex subunit with role in chromatin remodeling and NuA3 binding[67]
PRD1_5565	PRoteinase yscD[68]	Putative proteinase more highly expressed in clade I isolate with lower *MDR1* expression[69]	Zinc metalloproteinase[70] thought to be upregulated in DNA replication stress[71]
RCO1_4684	Rpd3S histone deacetylase complex[72]	-	Increased acetylation of *FLO8* and *STE11* in mutant *RCO1* in *C. cerevisiae*
RCO1_5541	”	-	”
SEC26_5544	SECretory[73]	Downregulated in amphotericin B resistant strains[52]	Protein trafficking from ER to golgi in *S. cerevisiae*[73]
TMA17_0329	Translation Machinery Associated[74]	-	Deletion associated with chromosome instability[75]
TMA17_5587	”	-	”

TRS33_5535	Trapp (transport protein particle) Subunit[76]	-	Require for required for Ypt1-mediated autophagy in *S. cerevisiae*[77]
YDL114W_5537	-	-	-

Table S2: Loci demonstrating copy number variation in clinical isolates.

Strain name	Anidulafungin EUCAST MIC mode (range, µg/mL)
StG1 Parent	2 (2-4)
StG1 SCV	2 (2-4)
StG1 LCV	8 (8-16)
K1 Parent	1 (1-2)
K1 SCV	1 (1-2)
K1 LCV	4 (2-8)

Table S3: EUCAST method antimicrobial testing for anidulafungin demonstrates elevated minimum inhibitory concentrations (MICs) in large colony variants (LCVs) compared to small colony variants (SCVs) and parent colonies derived from original clinical isolates.

162

Gene	Stands for	Function in *C. auris*	Function in other human fungal pathogens
ANT1_3678	Adenine Nucleotide Transporter[78]	-	Small molecule transport at peroxisome membrane[78]
APS2_1237	clathrin Associated Protein complex Small subunit	-	Cell membrane protein sorting in *S. cerevisiae*[79]
CAS1_1236	Caspofungin sensitivity[80]	-	Disruption confers caspofungin hypersensitivity in *C. albicans*[80], also known as ORF 19.1135 and a Ku70 orthologue, shown to regulate telomerase/recombination and modulate telomere length and structure[81]
CAT1_2298	Catalase[82]	-	Deletion in *C. albicans* has higher sensitivity to neutrophil ROS production and lower virulence[82]
CCT5_1136	Chaperonin Containing TCP-1[83]	-	Role in actin cytoskeleton organisation[83]
CRN1_4936	Coronin[84]	-	Actin cytoskeleton component[84]
CRZ1_2096	Calcineurin-Responsive Zinc-finger protein[3]	Predicted to play a role in β-glucan masking, putatively increasing immune escape and virulence[85]	Transcription factor driving *FKS* expression in *S. cerevisiae*[3], coordinating a broad range of stress responses via calcium and calcineurin pathways, including cell wall remodelling[4] including in azole tolerance[86] and echinocandin tolerance in *C. albicans*[87,88]. Deletion in *C. glabrata* confers echinocandin susceptibility, thus therapeutic targeting has been suggested[89]
GAT1_2524	GATA family transcription factor[90]	-	Regulator of nitrogen source metabolism[90]
GDA1_2126	Guanosine DiphosphatAse[91]	-	Mutants defective in O-mannosylation in *C. albicans*[92]
HOM3_3217	HOMoserine requiring[93]	-	Amino acid synthesis[93]
HOT13_4160	Helper of TIM[94]	-	Modifier of Translocase of Inner Mitochondrial Membrane[94]
HP_1698	Hypothetical protein	-	-
HP_2525	Hypothetical protein	-	-
HP_3898	Hypothetical protein	-	-
HP_4105	Hypothetical protein	-	-
HP_4897	Hypothetical protein	-	-
IFH1_2127	Interacts with Fork Head[95]	-	Regulator of ribosomal protein transcription[96]
LIA1_1135	Ligand of eIF5A[97]	-	Understood to be expressed in DNA replication stress[71]
LOS1_5427	Loss Of Suppression[98]	-	Nucleoskeleton component in *S. cerevisiae*[99]
MGT1_5291	O-6-MethylGuanine-DNA methylTransferase[100]	-	DNA repair methyltransferase; protection from DNA alkylating damage[100]
MUB1_3899	MUlti Budding[101]	Deletion increases Rpn4 levels leading to higher azole efflux pump expression inc. Cdr1[102]	Critical factor in Rpn4 ubiquitylation and degradation[103]
NOG2_0934	NucleOlar G-protein[104]	-	GTPase involved in ribosome maturation[104]
OFD1_3216	Prolyl 4-hydroxylase-like 2-Oxoglutarate-Fe(II) Dioxygenase[105]	-	Modulates morphology by Ume6 stabilisation in *C. albicans*[106]
PES1_3031	Pescadillo homolog[107]	-	Mutants defective in virulence; thought to be involved in morphology and lateral yeast growth in tissues in *C. albicans*[107]
PTC4_1697	Phosphatase Two C[108]	-	Mutation confers sensitivity to azoles; thought to be involved in mitochondrial ion homeostasis[109]

RAI1_2299	Rat1p Interacting Protein[110]	-	RNA processing and degradation[110]
RCN2_4159	Regulator of CalciNeurin[111]	-	Also part of calcineurin stress pathway[111]
SNU13_5510	Small NUclear ribonucleoprotein associated [[112]	-	RNA processing in *S. cerevisiae*[112]
STS1_2740	Sec Twenty-three Suppressor[113]	-	Proteasome targeting[114] and ribosomal RNA stability/protein transport roles in *S. cerevisiae*[113]
SUC2_4534	SUCrose[115]	-	Invertase[116]
TAZ1_3032	TAfaZzin[117]	-	Cell membrane phospholipid cardiolipin remodelling[117]
UTP7_2741	U Three Protein[118]	-	Ribosomal biogenesis[118]
YPR097W_0935	-	-	Modifies ergosterol distribution, also known as Lipid-droplet Ergosterol Cortex (LEC1)[119]

Table S4: Significantly variable loci and loci containing enriched terms between large (LCV) and small colony variants (SCVs). FDR: false discovery rate.

Gene	Stands for	Function in *C. auris*	Function in other human fungal pathogens
ACS1_3998	Acetyl-CoA synthetase[120]	-	Implicated in *C. albicans* carbon source range and virulence[121]
BCR1_3985	Biofilm and Cell wall Regulator[122]	Glycerol-induced Filamentation-Competent factor, mutations causing variations in filamentation competence and enhances skin colonisation with improved fatty acid β-oxidation metabolism[123]	Transcription factor regulating cell wall synthesis and biofilm formation[122,124]
COR1_3973	CORe protein of QH2 cytochrome c reductase[125]	-	Mitochondrial inner membrane electron transport chain component[125]
CSA1_1951	*Candida* Surface Antigen[126]	Upregulated in *ex vivo* whole blood model of infection[127]	GPI-anchored, CFEM-domain, hyphally located, Bcr1 regulated cell wall protein[124]
HP_0884	Hypothetical protein	-	-
HP_2234	Hypothetical protein	-	-
HP_2593	Hypothetical protein	24 nt deletion linked to pan-resistance strain[128]	-
HP_3931	Hypothetical protein	-	-
HP_3966	Hypothetical protein	-	Orthologous to transcription factor *Candida* Galactose gene Activator *CGA1*[129]
HP_4007	Hypothetical protein	-	-
IFF4_4451	Ipf family F[130]	Up-regulated in *C. auris* filamentation[131] and in ambisome-resistant strains[52], deletion has reduced adhesion[25], variants have been associated with echinocandin resistance in GWAS[132]	GPI-anchored, disruption reduces virulence/adherence in *C. albicans*[133]
NTO1_3949	NuA Three Orf[67]	-	Putative histone acetyltransferase (HAT) complex subunit with role in chromatin remodeling and NuA3 binding[67]
NUS1_2275	Nuclear Undecaprenyl pyrophosphate Synthase[134]	-	Regulation of filamentation, intracellular tracking and lipid homeostasis in *C. albicans*[6]
PGA6_1366	Predicted GPI-Anchored[135]	-	GPI-anchored[135]
RIB7_3941	Riboflavin biosynthesis[136]	-	Riboflavin synthesis across *Candida* species[137]
SPE4_3965	SPErmidine auxotroph[138]	-	-
WOR2_2136	White Opaque Regulator[139]	Under-expressed in *in vivo* catheter-associated biofilm[38]	Transcription factor implicated in *C. albicans* white-opaque switching[5]

Table S5: Loci with higher copy-number variation in large (LCV) or small colony variants (SCV) compared to clinical isolates.

Supplementary references

1. Burrack, L. S., Todd, R. T., Soisangwan, N., Wiederhold, N. P. & Selmecki, A. Genomic diversity across *Candida auris* clinical isolates shapes rapid development of antifungal resistance *in vitro* and *in vivo*. *mBio* **13**, e0084222 (2022).
2. Schikora-Tamarit, M. À. & Gabaldón, T. Recent gene selection and drug resistance underscore clinical adaptation across *Candida* species. *Nature Microbiology* **9**, 284–307 (2024).
3. Stathopoulos, A. M. & Cyert, M. S. Calcineurin acts through the *CRZ1/TCN1*-encoded transcription factor to regulate gene expression in yeast. *Genes & Development* **11**, 3432–3444 (1997).
4. Thewes, S. Calcineurin-Crz1 signaling in lower eukaryotes. *Eukaryotic Cell* **13**, 694–705 (2014).
5. Soll, D. R. The role of phenotypic switching in the basic biology and pathogenesis of *Candida albicans*. *Journal of Oral Microbiology* **6**, (2014).
6. Farheen, A. *et al.* The putative prenyltransferase Nus1 is required for filamentation in the human fungal pathogen *Candida albicans*. *G3* **14**, jkae124 (2024).
7. Munro, C. A. *et al.* The PKC, HOG and Ca2+ signalling pathways co-ordinately regulate chitin synthesis in *Candida albicans*. *Molecular microbiology* **63**, 1399–1413 (2007).
8. Walker, L. A. *et al.* Stimulation of chitin synthesis rescues *Candida albicans* from echinocandins. *PLOS pathogens* **4**, e1000040 (2008).
9. Wheeler, R. T., Kombe, D., Agarwala, S. D. & Fink, G. R. Dynamic, morphotype-specific *Candida albicans* beta-glucan exposure during infection and drug treatment. *PLOS pathogens* **4**, e1000227 (2008).
10. Perlin, D. S. Echinocandin resistance in *Candida*. *Clinical Infectious Diseases* **61**, S612–S617 (2015).
11. Widanage, M. C. D. *et al.* Unveiling cell wall structure and echinocandin response in *Candida auris* and *Candida albicans via* solid-state NMR. 2024.12.02.626508 (2024) doi:10.1101/2024.12.02.626508.

12. Singh-Babak, S. D. *et al.* Global analysis of the evolution and mechanism of echinocandin resistance in *Candida glabrata*. *PLOS Pathogens* **8**, e1002718 (2012).

13. Duxbury, S. J. N., Bates, S., Beardmore, R. E. & Gudelj, I. Evolution of drug-resistant and virulent small colonies in phenotypically diverse populations of the human fungal pathogen *Candida glabrata*. *Proceedings of the Royal Society B: Biological Sciences* **287**, 20200761 (2020).

14. Day, M. Yeast petites and small colony variants: For everything there is a season. *Advances in Applied Microbiology* **85**, 1–41 (2013).

15. Badrane, H. *et al.* Genotypic diversity and unrecognized antifungal resistance among populations of *Candida glabrata* from positive blood cultures. *Nature Communications* **14**, 5918 (2023).

16. Arastehfar, A. *et al.* Overlooked *Candida glabrata* petites are echinocandin tolerant, induce host inflammatory responses, and display poor *in vivo* fitness. *mBio* **14**, e01180–23 (2023).

17. Tscherner, M. *et al.* The *Candida albicans* histone acetyltransferase Hat1 regulates stress resistance and virulence via distinct chromatin assembly pathways. *PLOS pathogens* **11**, e1005218 (2015).

18. Li, X. *et al.* The Rpd3/Hda1 family of histone deacetylases regulates azole resistance in *Candida albicans*. *The Journal of Antimicrobial Chemotherapy* **70**, 1993–2003 (2015).

19. Chow, N. A. *et al.* Tracing the evolutionary history and global expansion of *Candida auris* using population genomic analyses. *mBio* **11**, 15 (2020).

20. Lockhart, S. R. *et al.* Simultaneous emergence of multidrug-resistant *Candida auris* on 3 continents confirmed by whole-genome sequencing and epidemiological analyses. *Clinical Infectious Diseases* **64**, 134–140 (2017).

21. Lee, K. S. & Levin, D. E. Dominant mutations in a gene encoding a putative protein kinase (*BCK1*) bypass the requirement for a *Saccharomyces cerevisiae* protein kinase C homolog. *Molecular and Cellular Biology* **12**, 172–182 (1992).

22. Lara-Aguilar, V. *et al.* Adaptation of the emerging pathogenic yeast *Candida auris* to high caspofungin concentrations correlates with cell wall changes. *Virulence* **12**, 1400–1417 (2021).

23. Blankenship, J. R., Fanning, S., Hamaker, J. J. & Mitchell, A. P. An extensive circuitry for cell wall regulation in *Candida albicans*. *PLOS Pathogens* **6**, e1000752 (2010).

24. Matsumoto, K., Uno, I., Oshima, Y. & Ishikawa, T. Isolation and characterization of yeast mutants deficient in adenylate cyclase and cAMP-dependent protein kinase. *Proceedings of the National Academy of Sciences of the United States of America* **79**, 2355–2359 (1982).

25. Santana, D. J. *et al.* A *Candida auris*-specific adhesin, Scf1, governs surface association, colonization, and virulence. *Science* **381**, 1461–1467 (2023).

26. Kim, J.-S., Lee, K.-T., Lee, M. H., Cheong, E. & Bahn, Y.-S. Adenylyl cyclase and protein kinase A play redundant and distinct roles in growth, differentiation, antifungal drug resistance, and pathogenicity of *Candida auris*. *mBio* **12**, e02729–21 (2021).

27. Ding, X., Cao, C., Zheng, Q. & Huang, G. The regulatory subunit of protein kinase A (Bcy1) in *Candida albicans* plays critical roles in filamentation and white-opaque switching but Is not essential for cell growth. *Frontiers in Microbiology* **7**, (2017).

28. Hartwell, L. H., Culotti, J. & Reid, B. Genetic control of the cell-division cycle in yeast, I. Detection of mutants. *Proceedings of the National Academy of Sciences of the United States of America* **66**, 352–359 (1970).

29. Vazquez, H. M., Vionnet, C., Roubaty, C. & Conzelmann, A. Cdc1 removes the ethanolamine phosphate of the first mannose of GPI anchors and thereby facilitates the integration of GPI proteins into the yeast cell wall. *Molecular Biology of the Cell* **25**, 3375–3388 (2014).

30. Uhl, M. A., Biery, M., Craig, N. & Johnson, A. D. Haploinsufficiency-based large-scale forward genetic analysis of filamentous growth in the diploid human fungal pathogen *C. albicans*. *The EMBO Journal* **22**, 2668–2678 (2003).

31. Garrett, M. D., Zahner, J. E., Cheney, C. M. & Novick, P. J. *GDI1* encodes a GDP dissociation inhibitor that plays an essential role in the yeast secretory pathway. *The EMBO journal* **13**, 1718–1728 (1994).

32. van Leeuwe, T. M., Gerritsen, A., Arentshorst, M., Punt, P. J. & Ram, A. F. J. Rab GDP-dissociation inhibitor *gdiA* is an essential gene required for cell wall chitin deposition in *Aspergillus niger*. *Fungal Genetics and Biology* **136**, 103319 (2020).

33. Mnaimneh, S. *et al.* Exploration of essential gene functions via titratable promoter alleles. *Cell* **118**, 31–44 (2004).

34. Yadav, A., Sah, S. K., Perlin, D. S. & Rustchenko, E. *Candida albicans* genes modulating echinocandin susceptibility of caspofungin-adapted mutants are constitutively expressed in clinical isolates with intermediate or full resistance to echinocandins. *Journal of Fungi* **10**, 224 (2024).

35. Fu, C. *et al.* Leveraging machine learning essentiality predictions and chemogenomic interactions to identify antifungal targets. *Nature Communications* **12**, 6497 (2021).

36. Lan, C.-Y. *et al.* Regulatory networks affected by iron availability in *Candida albicans*. *Molecular Microbiology* **53**, 1451–1469 (2004).

37. Smoak, R. A., Snyder, L. F., Fassler, J. S. & He, B. Z. Parallel expansion and divergence of an adhesin family in pathogenic yeasts. *Genetics* **223**, iyad024 (2023).

38. Wang, T. W. *et al.* Functional redundancy in *Candida auris* cell surface adhesins crucial for cell-cell interaction and aggregation. *Nature Communications* **15**, 9212 (2024).

39. Kean, R. *et al.* Transcriptome assembly and profiling of *Candida auris* reveals novel insights into biofilm-mediated resistance. *mSphere* **3**, e00334–18 (2018).

40. Dominguez, E. G. *et al.* Conserved role for biofilm matrix polysaccharides in *Candida auris* drug resistance. *mSphere* **4**, e00680–18 (2019).

41. Muñoz, J. F. *et al.* Clade-specific chromosomal rearrangements and loss of subtelomeric adhesins in *Candida auris*. *Genetics* **218**, iyab029 (2021).

42. Ford, C. B. *et al.* The evolution of drug resistance in clinical isolates of *Candida albicans*. *eLife* **4**, e00662 (2015).

43. Krogan, N. J. *et al.* Global landscape of protein complexes in the yeast *Saccharomyces* cerevisiae. *Nature* **440**, 637–643 (2006).

44. Lotz, H., Sohn, K., Brunner, H., Muhlschlegel, F. A. & Rupp, S. *RBR1*, a novel pH-regulated cell wall gene of *Candida albicans*, is repressed by *RIM101* and activated by *NRG1*. *Eukaryotic Cell* **3**, 776–784 (2004).

45. Tian, S. *et al.* Evolutionary accumulation of *FKS1* mutations from clinical echinocandin-resistant *Candida auris*. *Emerging Microbes & Infections* **13**, 2377584 (2024).

46. Boisramé, A., Cornu, A., Da Costa, G. & Richard, M. L. Unexpected role for a serine/threonine-rich domain in the *Candida albicans* Iff protein family. *Eukaryotic Cell* **10**, 1317–1330 (2011).

47. De Cesare, G. B., Hafez, A., Stead, D., Llorens, C. & Munro, C. A. Biomarkers of caspofungin resistance in *Candida albicans* isolates: A proteomic approach. *Virulence* **13**, 1005–1018 (2022).

48. Nogi, Y., Vu, L. & Nomura, M. An approach for isolation of mutants defective in 35S ribosomal RNA synthesis in *Saccharomyces cerevisiae*. *Proceedings of the National Academy of Sciences of the United States of America* **88**, 7026–7030 (1991).

49. Liu, T. T. *et al.* Genome-wide expression profiling of the response to azole, polyene, echinocandin, and pyrimidine antifungal agents in *Candida albicans*. *Antimicrobial Agents and Chemotherapy* **49**, 2226–2236 (2005).

50. Weil, T. *et al.* Adaptive mistranslation accelerates the evolution of fluconazole resistance and induces major genomic and gene expression alterations in *Candida albicans*. *mSphere* **2**, 10.1128/msphere.00167–17 (2017).

51. Fassler, J. S. & Winston, F. Isolation and analysis of a novel class of Suppressor of Ty insertion mutations in *Saccharomyces cerevisiae*. *Genetics* **118**, 203–212 (1988).

52. Shivarathri, R. *et al.* Comparative transcriptomics reveal possible mechanisms of amphotericin B resistance in *Candida auris*. *Antimicrobial Agents and Chemotherapy* **66**, e02276–21 (2022).

53. Kuchler, K., Jenull, S., Shivarathri, R. & Chauhan, N. Fungal KATs/KDACs: A new highway to better antifungal drugs? *PLOS Pathogens* **12**, e1005938 (2016).

54. Zheng, B., Wu, J. N., Schober, W., Lewis, D. E. & Vida, T. Isolation of yeast mutants defective for localization of vacuolar vital dyes. *Proceedings of the National Academy of Sciences of the United States of America* **95**, 11721–11726 (1998).

55. Robinson, J. S., Klionsky, D. J., Banta, L. M. & Emr, S. D. Protein sorting in *Saccharomyces cerevisiae*: Isolation of mutants defective in the delivery and processing of multiple vacuolar hydrolases. *Molecular and Cellular Biology* **8**, 4936–4948 (1988).

56. Spitzer, M. *et al.* Cross-species discovery of syncretic drug combinations that potentiate the antifungal fluconazole. *Molecular Systems Biology* **7**, 499 (2011).

57. Nissan, T. A., Baßler, J., Petfalski, E., Tollervey, D. & Hurt, E. 60S pre-ribosome formation viewed from assembly in the nucleolus until export to the cytoplasm. *The EMBO Journal* **21**, 5539–5547 (2002).

58. Yu, E. Y., Sun, J., Lei, M. & Lue, N. F. Analyses of *Candida* Cdc13 orthologues revealed a novel OB fold dimer arrangement, dimerization-assisted DNA binding, and substantial structural differences between Cdc13 and RPA70. *Molecular and Cellular Biology* **32**, 186–198 (2012).

59. Lussier, M. *et al.* Large scale identification of genes involved in cell surface biosynthesis and architecture in *Saccharomyces cerevisiae*. *Genetics* **147**, 435–450 (1997).

60. Lesage, G. *et al.* Analysis of beta-1,3-glucan assembly in *Saccharomyces cerevisiae* using a synthetic interaction network and altered sensitivity to caspofungin. *Genetics* **167**, 35–49 (2004).

61. Tournu, H. *et al.* Global role of the protein kinase Gcn2 in the human pathogen *Candida albicans*. *Eukaryotic Cell* **4**, 1687–1696 (2005).

62. Urban-Grimal, D. & Labbe-Bois, R. Genetic and biochemical characterization of mutants of *Saccharomyces cerevisiae* blocked in six different steps of heme biosynthesis. *Molecular and General Genetics MGG* **183**, 85–92 (1981).

63. Pais, P. *et al. Candida glabrata* transcription factor Rpn4 mediates fluconazole resistance through regulation of ergosterol biosynthesis and plasma membrane permeability. *Antimicrobial Agents and Chemotherapy* **64**, 10.1128/aac.00554–20 (2020).

64. Minaker, S. W., Filiatrault, M. C., Ben-Aroya, S., Hieter, P. & Stirling, P. C. Biogenesis of RNA polymerases II and III requires the conserved GPN small GTPases in *Saccharomyces cerevisiae*. *Genetics* **193**, 853–864 (2013).

65. Pel, H. J., Maat, C., Rep, M. & Grivell, L. A. The yeast nuclear gene *MRF1* encodes a mitochondrial peptide chain release factor and cures several mitochondrial RNA splicing defects. *Nucleic Acids Research* **20**, 6339–6346 (1992).

66. Fearon, K. & Mason, T. L. Structure and function of *MRP20* and *MRP49*, the nuclear genes for two proteins of the 54 S subunit of the yeast mitochondrial ribosome. *Journal of Biological Chemistry* **267**, 5162–5170 (1992).

67. Perry, J. The Epc-N domain: A predicted protein-protein interaction domain found in select chromatin associated proteins. *BMC Genomics* **7**, 6 (2006).

68. García-Alvarez, N., Teichert, U. & Wolf, D. H. Proteinase yscD mutants of yeast. Isolation and characterization. *European Journal of Biochemistry* **163**, 339–346 (1987).

69. Biermann, A. R. & Hogan, D. A. Transcriptional response of *Candida auris* to the Mrr1 inducers methylglyoxal and benomyl. *mSphere* (2022) doi:10.1101/2022.03.02.482751.

70. Büchler, M., Tisljar, U. & Wolf, D. H. Proteinase yscD (oligopeptidase yscD). Structure, function and relationship of the yeast enzyme with mammalian thimet oligopeptidase (metalloendopeptidase, EP 24.15). *European Journal of Biochemistry* **219**, 627–639 (1994).

71. Tkach, J. M. *et al.* Dissecting DNA damage response pathways by analysing protein localization and abundance changes during DNA replication stress. *Nature Cell Biology* **14**, 966–976 (2012).

72. Carrozza, M. J. *et al.* Histone H3 methylation by Set2 directs deacetylation of coding regions by Rpd3S to suppress spurious intragenic transcription. *Cell* **123**, 581–592 (2005).

73. Duden, R. *et al.* Yeast beta- and beta'-coat proteins (COP). Two coatomer subunits essential for endoplasmic reticulum-to-Golgi protein traffic. *Journal of Biological Chemistry* **269**, 24486–24495 (1994).

74. Fleischer, T. C., Weaver, C. M., McAfee, K. J., Jennings, J. L. & Link, A. J. Systematic identification and functional screens of uncharacterized proteins associated with eukaryotic ribosomal complexes. *Genes & Development* **20**, 1294–1307 (2006).

75. Strome, E. D., Wu, X., Kimmel, M. & Plon, S. E. Heterozygous screen in *Saccharomyces cerevisiae* identifies dosage-sensitive genes that affect chromosome stability. *Genetics* **178**, 1193–1207 (2008).

76. Sacher, M., Barrowman, J., Schieltz, D., Yates, J. R. & Ferro-Novick, S. Identification and characterization of five new subunits of TRAPP. *European Journal of Cell Biology* **79**, 71–80 (2000).

77. Lipatova, Z., Majumdar, U. & Segev, N. Trs33-containing TRAPP IV: A novel autophagy-specific Ypt1 GEF. *Genetics* **204**, 1117–1128 (2016).

78. Palmieri, L. *et al.* Identification and functional reconstitution of the yeast peroxisomal adenine nucleotide transporter. *The EMBO Journal* **20**, 5049–5059 (2001).

79. Phan, H. L. *et al.* The *Saccharomyces cerevisiae APS1* gene encodes a homolog of the small subunit of the mammalian clathrin AP-1 complex: Evidence for functional interaction with clathrin at the Golgi complex. *The EMBO journal* **13**, 1706–1717 (1994).

80. Bruno, V. M. *et al.* Control of the *C. albicans* cell wall damage response by transcriptional regulator Cas5. *PLOS Pathogens* **2**, e21 (2006).

81. Chico, L., Ciudad, T., Hsu, M., Lue, N. F. & Larriba, G. The *Candida albicans* Ku70 Modulates Telomere Length and Structure by Regulating Both Telomerase and Recombination. *PLOS ONE* **6**, e23732 (2011).

82. Wysong, D. R., Christin, L., Sugar, A. M., Robbins, P. W. & Diamond, R. D. Cloning and sequencing of a *Candida albicans* catalase gene and effects of disruption of this gene. *Infection and Immunity* **66**, 1953–1961 (1998).

83. Vinh, D. B. & Drubin, D. G. A yeast TCP-1-like protein is required for actin function in vivo. *Proceedings of the National Academy of Sciences of the United States of America* **91**, 9116–9120 (1994).

84. Heil-Chapdelaine, R. A., Tran, N. K. & Cooper, J. A. The role of *Saccharomyces cerevisiae* coronin in the actin and microtubule cytoskeletons. *Current biology: CB* **8**, 1281–1284 (1998).

85. Selisana, S. M. G. *et al.* Alteration of β-glucan in the emerging fungal pathogen *Candida auris* leads to immune evasion and increased virulence. *Medical Microbiology and Immunology* **213**, 13 (2024).

86. Onyewu, C., Wormley, F. L., Perfect, J. R. & Heitman, J. The calcineurin target, Crz1, functions in azole tolerance but is not required for virulence of *Candida albicans*. *Infection and Immunity* **72**, 7330–7333 (2004).

87. Singh, S. D. *et al.* Hsp90 governs echinocandin resistance in the pathogenic yeast *Candida albicans* via calcineurin. *PLOS pathogens* **5**, e1000532 (2009).

88. Walker, L. A., Gow, N. A. R. & Munro, C. A. Fungal echinocandin resistance. *Fungal Genetics and Biology* **47**, 117–126 (2010).

89. Ceballos-Garzon, A. *et al.* CRISPR-Cas9 approach confirms Calcineurin-responsive zinc finger 1 (Crz1) transcription factor as a promising therapeutic target in echinocandin-resistant *Candida glabrata*. *PLoS One* **17**, e0265777 (2022).

90. Coffman, J. A., Rai, R., Cunningham, T., Svetlov, V. & Cooper, T. G. Gat1p, a GATA family protein whose production is sensitive to nitrogen catabolite repression, participates in transcriptional activation of nitrogen-catabolic genes in *Saccharomyces cerevisiae*. *Molecular and Cellular Biology* **16**, 847–858 (1996).

91. Berninsone, P., Miret, J. J. & Hirschberg, C. B. The Golgi guanosine diphosphatase is required for transport of GDP-mannose into the lumen of *Saccharomyces cerevisiae* Golgi vesicles. *The Journal of Biological Chemistry* **269**, 207–211 (1994).

92. Herrero, A. B., Uccelletti, D., Hirschberg, C. B., Dominguez, A. & Abeijon, C. The golgi GDPase of the fungal pathogen *Candida albicans* affects morphogenesis, glycosylation, and cell wall properties. *Eukaryotic Cell* **1**, 420–431 (2002).

93. Rafalski, J. A. & Falco, S. C. Structure of the yeast *HOM3* gene which encodes aspartokinase. *The Journal of Biological Chemistry* **263**, 2146–2151 (1988).

94. Curran, S. P. *et al.* The role of Hot13p and redox chemistry in the mitochondrial TIM22 import pathway. *Journal of Biological Chemistry* **279**, 43744–43751 (2004).

95. Cherel, I. & Thuriaux, P. The *IFH1* gene product interacts with a fork head protein in *Saccharomyces cerevisiae*. *Yeast (Chichester, England)* **11**, 261–270 (1995).

96. Mallick, J. & Whiteway, M. The evolutionary rewiring of the ribosomal protein transcription pathway modifies the interaction of transcription factor heteromer Ifh1-Fhl1 (interacts with forkhead 1-forkhead-like 1) with the DNA-binding specificity element. *The Journal of Biological Chemistry* **288**, 17508–17519 (2013).

97. Thompson, G. M., Cano, V. S. P. & Valentini, S. R. Mapping eIF5A binding sites for Dys1 and Lia1: *in vivo* evidence for regulation of eIF5A hypusination. *FEBS letters* **555**, 464–468 (2003).

98. Hopper, A. K., Schultz, L. D. & Shapiro, R. A. Processing of intervening sequences: A new yeast mutant which fails to excise intervening sequences from precursor tRNAs. *Cell* **19**, 741–751 (1980).

99. Shen, W. C., Selvakumar, D., Stanford, D. R. & Hopper, A. K. The *Saccharomyces cerevisiae* LOS1 gene involved in pre-tRNA splicing encodes a nuclear protein that behaves as a component of the nuclear matrix. *The Journal of Biological Chemistry* **268**, 19436–19444 (1993).

100. Xiao, W., Derfler, B., Chen, J. & Samson, L. Primary sequence and biological functions of a *Saccharomyces cerevisiae* O6-methylguanine/O4-methylthymine DNA repair methyltransferase gene. *The EMBO journal* **10**, 2179–2186 (1991).

101. Krüger, M. & Fischer, R. Integrity of a Zn finger-like domain in SamB is crucial for morphogenesis in ascomycetous fungi. *The EMBO journal* **17**, 204–214 (1998).

102. Chow, E. W. L. *et al.* The transcription factor Rpn4 activates its own transcription and induces efflux pump expression to confer fluconazole resistance in *Candida auris*. *mBio* **14**, e0268823 (2023).

103. Ju, D., Wang, X., Xu, H. & Xie, Y. Genome-wide analysis identifies MYND-domain protein Mub1 as an essential factor for Rpn4 ubiquitylation. *Molecular and Cellular Biology* **28**, 1404–1412 (2008).

104. Saveanu, C. *et al.* Nog2p, a putative GTPase associated with pre-60S subunits and required for late 60S maturation steps. *The EMBO Journal* **20**, 6475–6484 (2001).

105. Hughes, B. T. & Espenshade, P. J. Oxygen-regulated degradation of fission yeast SREBP by Ofd1, a prolyl hydroxylase family member. *The EMBO Journal* **27**, 1491–1501 (2008).

106. Lu, Y., Su, C., Solis, N. V., Filler, S. G. & Liu, H. Synergistic regulation of hyphal elongation by hypoxia, CO(2), and nutrient conditions controls the virulence of *Candida albicans*. *Cell Host & Microbe* **14**, 499–509 (2013).

107. Uppuluri, P. *et al.* Physiologic expression of the *Candida albicans* pescadillo homolog is required for virulence in a murine model of hematogenously disseminated candidiasis. *Eukaryotic Cell* **11**, 1552–1556 (2012).

108. Cheng, A., Ross, K. E., Kaldis, P. & Solomon, M. J. Dephosphorylation of cyclin-dependent kinases by type 2C protein phosphatases. *Genes & Development* **13**, 2946–2957 (1999).

109. Zhao, J. *et al.* Identification and characterization of the type 2C protein phosphatase Ptc4p in the human fungal pathogen *Candida albicans*. *Yeast (Chichester, England)* **27**, 149–157 (2010).

110. Xue, Y. *et al. Saccharomyces cerevisiae* RAI1 (YGL246c) is homologous to human DOM3Z and encodes a protein that binds the nuclear exoribonuclease Rat1p. *Molecular and Cellular Biology* **20**, 4006–4015 (2000).

111. Mehta, S., Li, H., Hogan, P. G. & Cunningham, K. W. Domain architecture of the regulators of calcineurin (RCANs) and identification of a divergent RCAN in yeast. *Molecular and Cellular Biology* **29**, 2777–2793 (2009).

112. Stevens, S. W. & Abelson, J. Purification of the yeast U4/U6·U5 small nuclear ribonucleoprotein particle and identification of its proteins. *Proceedings of the National Academy of Sciences of the United States of America* **96**, 7226–7231 (1999).

113. Liang, S., Lacroute, F. & Képès, F. Multicopy STS1 restores both protein transport and ribosomal RNA stability in a new yeast Sec23 mutant allele. *European Journal of Cell Biology* **62**, 270–281 (1993).

114. Chen, L. *et al.* Sts1 plays a key role in targeting proteasomes to the nucleus. *The Journal of Biological Chemistry* **286**, 3104–3118 (2011).

115. Carlson, M., Osmond, B. C. & Botstein, D. Mutants of yeast defective in sucrose utilization. *Genetics* **98**, 25–40 (1981).

116. Lutfiyya, L. L. & Johnston, M. Two zinc-finger-containing repressors are responsible for glucose repression of *SUC2* expression. *Molecular and Cellular Biology* **16**, 4790–4797 (1996).

117. Gu, Z. *et al.* Aberrant cardiolipin metabolism in the yeast Taz1 mutant: A model for Barth syndrome. *Molecular Microbiology* **51**, 149–158 (2004).

118. Dragon, F. *et al.* A large nucleolar U3 ribonucleoprotein required for 18S ribosomal RNA biogenesis. *Nature* **417**, 967–970 (2002).

119. Castro, I. G. *et al.* Systematic analysis of membrane contact sites in *Saccharomyces cerevisiae* uncovers modulators of cellular lipid distribution. *eLife* **11**, e74602 (2022).

120. De Virgilio, C. *et al.* Cloning and disruption of a gene required for growth on acetate but not on ethanol: The acetyl-coenzyme A synthetase gene of *Saccharomyces cerevisiae*. *Yeast (Chichester, England)* **8**, 1043–1051 (1992).

121. Carman, A. J., Vylkova, S. & Lorenz, M. C. Role of acetyl coenzyme A synthesis and breakdown in alternative carbon source utilization in *Candida albicans*. *Eukaryotic Cell* **7**, 1733–1741 (2008).

122. Nobile, C. J. & Mitchell, A. P. Regulation of cell-surface genes and biofilm formation by the *C. albicans* transcription factor Bcr1p. *Current biology: CB* **15**, 1150–1155 (2005).

123. Deng, Y. *et al.* A single gene mutation underpins metabolic adaptation and acquisition of filamentous competence in the emerging fungal pathogen *Candida auris*. *PLOS Pathogens* **20**, e1012362 (2024).

124. Srikantha, T., Daniels, K. J., Pujol, C., Kim, E. & Soll, D. R. Identification of genes upregulated by the transcription factor Bcr1 that are involved in impermeability, impenetrability, and drug resistance of *Candida albicans* a/α biofilms. *Eukaryotic Cell* **12**, 875–888 (2013).

125. Tzagoloff, A., Wu, M. A. & Crivellone, M. Assembly of the mitochondrial membrane system. Characterization of *COR1*, the structural gene for the 44-kilodalton

core protein of yeast coenzyme QH2-cytochrome c reductase. *The Journal of Biological Chemistry* **261**, 17163–17169 (1986).

126. Lamarre, C., Deslauriers, N. & Bourbonnais, Y. Expression cloning of the *Candida albicans CSA1* gene encoding a mycelial surface antigen by sorting of *Saccharomyces cerevisiae* transformants with monoclonal antibody-coated magnetic beads. *Molecular Microbiology* **35**, 444–453 (2000).

127. Allert, S. *et al.* From environmental adaptation to host survival: Attributes that mediate pathogenicity of *Candida auris*. *Virulence* **13**, 191–214 (2022).

128. Rhodes, J. *et al.* What makes *Candida auris* pan-drug resistant? Integrative insights from genomic, transcriptomic, and phenomic analysis of clinical strains resistant to all four major classes of antifungal drugs. *Antimicrobial Agents and Chemotherapy* **68**, e00911–24 (2024).

129. Sun, X. *et al.* Recognition of galactose by a scaffold protein recruits a transcriptional activator for the GAL regulon induction in *Candida albicans*. *eLife* **12**, e84155 (2023).

130. Bates, S. *et al. Candida albicans* Iff11, a secreted protein required for cell wall structure and virulence. *Infection and Immunity* **75**, 2922–2928 (2007).

131. Kim, S. H. *et al.* Genetic analysis of *Candida auris* implicates Hsp90 in morphogenesis and azole tolerance and Cdr1 in Azole Resistance. *mBio* **10**, e02529–18 (2019).

132. Wang, Y. & Xu, J. Associations between genomic variants and antifungal susceptibilities in the archived global *Candida auris* population. *Journal of Fungi* **10**, 86 (2024).

133. Kempf, M. *et al.* Disruption of the GPI protein-encoding gene *IFF4* of *Candida albicans* results in decreased adherence and virulence. *Mycopathologia* **168**, 73–77 (2009).

134. Cherry, J. M. The *Saccharomyces* Genome Database: A tool for discovery. *Cold Spring Harbor Protocols* **2015**, pdb.top083840 (2015).

135. Plaine, A. *et al.* Functional analysis of *Candida albicans* GPI-anchored proteins: Roles in cell wall integrity and caspofungin sensitivity. *Fungal Genetics and Biology* **45**, 1404–1414 (2008).

136. Oltmanns, O. & Bacher, A. Biosynthesis of riboflavine in *Saccharomyces cerevisiae*: The role of genes *RIB1* and *RIB7*. *Journal of Bacteriology* **110**, 818–822 (1972).

137. Nysten, J. *et al.* The riboflavin biosynthetic pathway as a novel target for antifungal drugs against *Candida* species. *mBio* **15**, e0250224 (2024).

138. Whitney, P. A. & Morris, D. R. Polyamine auxotrophs of *Saccharomyces cerevisiae*. *Journal of Bacteriology* **134**, 214–220 (1978).

139. Zordan, R. E., Miller, M. G., Galgoczy, D. J., Tuch, B. B. & Johnson, A. D. Interlocking transcriptional feedback loops control white-opaque switching in *Candida albicans*. *PLOS Biology* **5**, e256 (2007).
