# Supplementary material for: FKS1/2-variant independent mechanisms underlying the emergence of resistance in echinocandin-refractory Candida auris infections
Source: Nat Commun. 2026 Oct 15;17:10406. doi: 10.1038/s41467-026-76954-x (PMC13672790; doi:10.1038/s41467-026-76954-x)
Supplement: Supplementary file 2 [file 41467_2026_76954_MOESM2_ESM.pdf]

nature portfolio

Corresponding author(s): Hugh Gifford

Last updated by author(s): 22/07/2026

Reporting Summary

Nature Portfolio wishes to improve the reproducibility of the work that we publish. This form provides structure for consistency and transparency in reporting. For further information on Nature Portfolio policies, see our Editorial Policies and the Editorial Policy Checklist.

Statistics

For all statistical analyses, confirm that the following items are present in the figure legend, table legend, main text, or Methods section.

n/a	Confirmed	
☐	☒	The exact sample size (*n*) for each experimental group/condition, given as a discrete number and unit of measurement
☐	☒	A statement on whether measurements were taken from distinct samples or whether the same sample was measured repeatedly
☐	☒	The statistical test(s) used AND whether they are one- or two-sided<br>*Only common tests should be described solely by name; describe more complex techniques in the Methods section.*
☒	☐	A description of all covariates tested
☐	☒	A description of any assumptions or corrections, such as tests of normality and adjustment for multiple comparisons
☐	☒	A full description of the statistical parameters including central tendency (e.g. means) or other basic estimates (e.g. regression coefficient) AND variation (e.g. standard deviation) or associated estimates of uncertainty (e.g. confidence intervals)
☐	☒	For null hypothesis testing, the test statistic (e.g. *F*, *t*, *r*) with confidence intervals, effect sizes, degrees of freedom and *P* value noted<br>*Give P values as exact values whenever suitable.*
☒	☐	For Bayesian analysis, information on the choice of priors and Markov chain Monte Carlo settings
☒	☐	For hierarchical and complex designs, identification of the appropriate level for tests and full reporting of outcomes
☒	☐	Estimates of effect sizes (e.g. Cohen's *d*, Pearson's *r*), indicating how they were calculated

Our web collection on statistics for biologists contains articles on many of the points above.

Software and code

Policy information about availability of computer code

Data collection: Data was inputted manually into Microsoft Excel for Mac v16.90 and handled within RStudio 2024.04.2+764.

Data analysis: No data were excluded from the analyses.

Variant Calling was performed using the Genome Analysis Toolkit (GATK) v4.1.2.0 with alignment of raw sequences to the B8441 v2 reference genome (GCA_002759435.2) and the mitochondrial genome (NC_053321.1) using BWA-MEM v0.7.17. HaplotypeCaller was executed in GVCF mode with the haploid ploidy flag. Hard filters were used to remove spurious variants, including the filters quality by depth (QD) <2.0, Fisher Strand (FS) >60.0 and root mean square mapping quality (MQ) <40.0.

Colony sizes were measured manually using ImageJ at each timepoint. Briefly, images were converted to black and white, sharpened, and then threshold changed to remove parts of the plate with no colony growing. Plots represent distribution of all colony sizes from 3 biological replicates for each cell type. One-way analysis of variance (ANOVA) was used.

OD600 was measured using a Spectrostar Nano every 10 min. for 50 h. SMG was determined as average 72 h growth in the wells above MIC divided by the growth in the no-drug well.

FastTree v2.1.11 with Jukes-Cantor modelling was used to construct phylogenetic trees based on multiple sequence FASTA alignments produced by ECATools using default parameters; midpoint rooted trees used 1,015 (StG clinical series plus laboratory evolved daughters) or 1,053 (K clinical series plus laboratory evolved daughters) phylogenetically informative sites. Additional sequences used to contextualise clinical samples within clades were obtained from the largest global genomic epidemiology study to date and the first five isolates were called

and used for phylogenetic reconstruction, for which 192,032 sites were entirely covered in all.

Copy number variation estimation and depth-of-coverage plots were calculated in comparison to normalised depth of coverage across the whole genome compared to the depth of coverage across either all positions for each gene locus or each 10 kb sliding window, from pileups created with Samtools mpileup. Significance testing for variants/loci/functional annotations was performed using Fisher's exact test (two-sided) with Benjamini-Hochberg (BH) testing for multiple correction with a cut-off of 0.05. Significance testing for copy number variation was performed with Student's T-test (two-sided), also with BH testing, but with a cut-off FDR of 0.005. Functional annotations and gene names were imported from prior analyses as described previously. To identify the promoter site for Crz1 binding upstream of FKS1, we used Yeastract+ to ascertain the presence of GNGGCKCA at contig PEKT02000002.1 position 1009439-1009439 (893-900 bases upstream from FKS1 start) as the putative binding site for Crz1 (GAGGCCGCA). Contig edges were inferred using the B8441 v3 assembly, which demonstrates seven contigs. To test for polyploidy, variants were re-called using GATK with the diploid flag to count heterozygous positions per 10 kb. Allele frequency counts were calculated from mpileups derived as above.

The chitin content in the cell walls of C. auris isolates and their segregants was compared using previously described methods. Cells were grown in YPD at 30 °C for 5 h with or without 8 μg/ml anidulafungin, fixed with 50 mM thimerosal, and stained with 10 μg/mL Calcofluor-White (CFW) in the dark for 60 min. Stained cells were washed twice with PBS, and their fluorescence quantified using an Attune NxT flow cytometer. The plots represent three biological replicate experiments, in each of which 10,000 events were acquired. As a negative control, cells were treated as above but without the addition of CFW. Median Fluorescence Intensities (MFI) were determined using FlowJo v.10 software. The exposure of β-glucan at the C. auris cell surface was quantified by flow cytometry as described previously. Cells were fixed overnight with thimerosal and stained with Fc-Dectin-1 and anti-human IgG linked to Alexafluor 488 (Jackson ImmunoResearch, Ely, UK). The fluorescence of 10,000 cells per condition was assayed using an Attune NxT flow cytometer. Median Fluorescence Intensity (MFI) was quantified using FlowJo v.10 software, and fold changes in β-1,3-glucan exposure were calculated relative to the control.

Transmission electron microscopy (TEM) was performed as described previously. Cells were subjected to high pressure freezing and freeze substitution, and fixed and stained with 1% osmium tetroxide and 0.5% glutaraldehyde. Ultrathin sections (60 nm) were prepared using lead citrate for contrast and imaged (JEOL 1400 JEM transmission electron microscope with ES1000W Gatan CCD camera). Cell wall sections were imaged at a nominal magnification of x100k. For each condition 10 measurements of inner and outer cell wall diameter were taken for each cell using the line tool in ImageJ (n = 33, StG1 parent; n =29, SCV -ANF; n =53, SCV +ANF; n =35, LCV -ANF; n = 43, LCV +ANF) and were analysed using two-sided Brown-Forsythe and Welch ANOVA.

Determination of cell wall mannan, chitin, and b-glucan content was achieved by acid hydrolysing the polymers, and quantifying mannose, glucosamine, and glucose content, respectively, by high-performance anion-exchange ion chromatography with pulsed amperometric detection (HPIC) as previously described.

The ploidy of cells in C. auris colonies growing on YPD plates containing 8 μg/ml anidulafungin was assayed by flow cytometry using previously described procedures. Before flow cytometry, samples were sonicated and washed with PBS. Flow cytometry was performed on an Attune NxT flow cytometer using an excitation wavelength of 488 nm. SYBR Green I fluorescence was detected with a 530/30 band pass filter and 50,000 events, gated for single cells, were recorded for each sample. The proportions of individual cells that displayed ploidies of 1n, 2n, 3n, 4n or >5n were quantified using FlowJo v.10 software, using isogenic haploid and diploid colonies of S. cerevisiae grown on YPD plates as controls: W303-1B a (MATa, ade2, his3, leu2, trp1, ura3); W303-1B 2M (MATa/MATα, ade2/ade2, his3/his3, leu2/leu2, trp1/trp1, ura3/ura3). The cells were also examined by fluorescence microscopy, staining them as described above with the addition of CFW (25 μg/ml) for 5 min. Cells were imaged using a DeltaVision Elite fluorescence microscope with a 60 x objective. Fluorescence excitation was generated by a LumencorLED light source and 10 μm Z-stacks of 50 images were captured by a pco.edge sCMOScamera. The 3D stacks were then deconvolved to remove out of focus light and maximum intensity projections used to create a 2D image by Image-J v1.54.

For manuscripts utilizing custom algorithms or software that are central to the research but not yet described in published literature, software must be made available to editors and reviewers. We strongly encourage code deposition in a community repository (e.g. GitHub). See the Nature Portfolio guidelines for submitting code & software for further information.

Data

Policy information about availability of data

All manuscripts must include a data availability statement. This statement should provide the following information, where applicable:

- Accession codes, unique identifiers, or web links for publicly available datasets
- A description of any restrictions on data availability
- For clinical datasets or third party data, please ensure that the statement adheres to our policy

Raw reads have been deposited via NCBI. Data availability statement is included.

Research involving human participants, their data, or biological material

Policy information about studies with human participants or human data. See also policy information about sex, gender (identity/presentation), and sexual orientation and race, ethnicity and racism.

Reporting on sex and gender	NA
Reporting on race, ethnicity, or other socially relevant groupings	NA
Population characteristics	NA
Recruitment	NA

Ethics oversight	NA

Note that full information on the approval of the study protocol must also be provided in the manuscript.

Field-specific reporting

Please select the one below that is the best fit for your research. If you are not sure, read the appropriate sections before making your selection.

☒ Life sciences ☐ Behavioural & social sciences ☐ Ecological, evolutionary & environmental sciences

For a reference copy of the document with all sections, see nature.com/documents/nr-reporting-summary-flat.pdf

Life sciences study design

All studies must disclose on these points even when the disclosure is negative.

Sample size	See data analysis section above.
Data exclusions	See data analysis section above.
Replication	NA
Randomization	NA
Blinding	NA

Reporting for specific materials, systems and methods

We require information from authors about some types of materials, experimental systems and methods used in many studies. Here, indicate whether each material, system or method listed is relevant to your study. If you are not sure if a list item applies to your research, read the appropriate section before selecting a response.

Materials & experimental systems

n/a	Involved in the study	
☒	☐	Antibodies
☒	☐	Eukaryotic cell lines
☒	☐	Palaeontology and archaeology
☒	☐	Animals and other organisms
☒	☐	Clinical data
☒	☐	Dual use research of concern
☒	☐	Plants

Methods

n/a	Involved in the study	
☒	☐	ChIP-seq
☒	☐	Flow cytometry
☒	☐	MRI-based neuroimaging

Plants

Seed stocks	NA
Novel plant genotypes	NA
Authentication	NA
