# Supplementary material for: FKS1/2-variant independent mechanisms underlying the emergence of resistance in echinocandin-refractory Candida auris infections
Source: Nat Commun. 2026 Oct 15;17:10406. doi: 10.1038/s41467-026-76954-x (PMC13672790; doi:10.1038/s41467-026-76954-x)
Supplement: Supplementary file 3 [file 41467_2026_76954_MOESM3_ESM.pdf]

nature portfolio

Peer Review File

FKS1/2-variant independent mechanisms underlying the emergence of resistance in echinocandin-refractory *Candida auris* infections

Corresponding Author: Dr Hugh Gifford

This file contains all reviewer reports in order by version, followed by all author rebuttals in order by version.

Version 0:

Reviewer comments:

Reviewer #1

(Remarks to the Author)
This manuscript presents a highly engaging and meticulously conducted study on novel in vitro mechanisms of echinocandin resistance in Candida auris. The work is scientifically robust, elegantly articulated and provides important conceptual refinements to our understanding of antifungal resistance.
The authors convincingly demonstrate that tolerance, heteroresistance and anidulafungin resistance may arise in association with genetic alterations beyond the FKS loci, and through states of heteroploidy.
Their proposed update to the three stage model of echinocandin resistance development in Candida is both insightful and intellectually compelling. In this framework, resistance is postulated to originate from a reversible state of echinocandin tolerance driven by physiological adaptations; subsequent sub MIC drug exposure favours the gradual emergence of more resistant phenotypes; and antifungal induced increases in ploidy facilitate the accumulation of escape mutations that are ultimately selected under sustained drug pressure.
Overall, the manuscript is of very high quality, and I consider it a valuable contribution to the field. I do not identify any issues warranting major revision. Nevertheless, I believe the clarity and precision of the manuscript could be further enhanced through attention to a number of minor points, which I outline below with reference to the corresponding line numbers.

Minor Comments and Recommendations:
Lines 52–53
Please revise “after detection in Japan in 2008” to “after description in Japan in 2008”, as earlier clinical isolates were retrospectively identified in South Korea and other regions.
Lines 55–56
In the sentence referring to the challenges of eradicating C. auris from hospital environments, it would be appropriate to include explicit mention of the role of fomites and contaminated surfaces in facilitating its persistence and transmission.
Lines 57–58
As C. auris infections have now been reported across all continents except Antarctica, it may be preferable to remove continental descriptors. The sentence could be reformulated as:
“Colonisation and infection rates continue to rise (14, 15, 16, 17, 18, plus an additional reference).”
I respectfully suggest including the following citation:
Lane CR, Seemann T, Worth LJ, Easton M, Pitchers W, Wong J, Cameron D, Azzato F, Bartolo R, Mateevici C, Marshall C, Slavin MA, Howden BP, Williamson DA. Incursions of Candida auris into Australia, 2018. Emerg Infect Dis. 2020;26(6):1326-1328. doi: 10.3201/eid2606.190936.
Line 65
Replace “uncommon” with “low”, as percentages in the 0–9% range are more accurately characterised in this manner.
Line 71
Replace “murine infections models” with “murine candidiasis model”.
Lines 81–83
The authors may wish to clarify whether the term “strains” or “clinical isolates” is most appropriate in the context of the mutations described.
Lines 90–93
Replace “…has been reported in patients receiving treatment…” with “…has been reported in isolates from patients

receiving treatment…" to maintain precision.
Line 100
Replace with the fuller formulation:
"Candida parapsilosis, Candida glabrata (also named Nakaseomyces glabratus) and C. albicans. Heteroresistance…"
Lines 113–115
Replace "C. auris bloodstream infections" with "C. auris candidaemia", consistent with the terminology used in Figure 1.
Line 124
Replace "two patients with bloodstream infections (BSI)" with "two patients with candidaemia".
Line 128
Replace "initial BSI" with "initial candidaemia".
Line 133
Replace "the fungal infection" with "the candidiasis".
Line 173
Replace "echinocandin sensitive" with "echinocandin susceptible".
Lines 302–303
Replace "echinocandin sensitivity" with "echinocandin susceptibility".
Lines 363–364
Replace "host immunosuppression" with "host immunodeficiency", as critically ill patients may present with variable degrees of immune dysfunction, not necessarily resulting from immunosuppression.
Lines 371–372
Please include a link or bibliographic reference to the Wellcome Trust funded COMBAT Candida clinical trial.

Reviewer #2

(Remarks to the Author)

Version 1:

Reviewer comments:

Reviewer #2

(Remarks to the Author)

Open Access This Peer Review File is licensed under a Creative Commons Attribution 4.0 International License, which permits use, sharing, adaptation, distribution and reproduction in any medium or format, as long as you give appropriate credit to the original author(s) and the source, provide a link to the Creative Commons license, and indicate if changes were made.
In cases where reviewers are anonymous, credit should be given to 'Anonymous Referee' and the source.
The images or other third party material in this Peer Review File are included in the article's Creative Commons license,

unless indicated otherwise in a credit line to the material. If material is not included in the article's Creative Commons license and your intended use is not permitted by statutory regulation or exceeds the permitted use, you will need to obtain permission directly from the copyright holder.
To view a copy of this license, visit https://creativecommons.org/licenses/by/4.0/

RESPONSE TO REVIEWS

Reviewer 1

This manuscript presents a highly engaging and meticulously conducted study on novel *in vitro* mechanisms of echinocandin resistance in *Candida auris*. The work is scientifically robust, elegantly articulated and provides important conceptual refinements to our understanding of antifungal resistance. The authors convincingly demonstrate that tolerance, heteroresistance and anidulafungin resistance may arise in association with genetic alterations beyond the *FKS* loci, and through states of heteroploidy. Their proposed update to the three stage model of echinocandin resistance development in *Candida* is both insightful and intellectually compelling. In this framework, resistance is postulated to originate from a reversible state of echinocandin tolerance driven by physiological adaptations; subsequent sub MIC drug exposure favours the gradual emergence of more resistant phenotypes; and antifungal induced increases in ploidy facilitate the accumulation of escape mutations that are ultimately selected under sustained drug pressure. Overall, the manuscript is of very high quality, and I consider it a valuable contribution to the field. I do not identify any issues warranting major revision. Nevertheless, I believe the clarity and precision of the manuscript could be further enhanced through attention to a number of minor points, which I outline below with reference to the corresponding line numbers.

Many thanks for this positive feedback. We are very grateful.

Minor Comments and Recommendations:
Lines 52–53
Please revise “after detection in Japan in 2008” to “after description in Japan in 2008”, as earlier clinical isolates were retrospectively identified in South Korea and other regions.

Thank you for pointing out this clarification. We now state that the description occurred in 2008, before publication in 2009.

Lines 55–56
In the sentence referring to the challenges of eradicating *C. auris* from hospital environments, it would be appropriate to include explicit mention of the role of fomites and contaminated surfaces in facilitating its persistence and transmission.

We have rephrased this sentence accordingly, stating “Furthermore, *C. auris* is challenging to eradicate from hospital environments owing to its persistence on abiotic surfaces, such as contaminated surfaces and hospital equipment acting as fomites, resulting in inter-patient spread within and between hospitals” (now lines 55-57).

Lines 57–58
As *C. auris* infections have now been reported across all continents except Antarctica, it may be preferable to remove continental descriptors. The sentence could be reformulated as:

"Colonisation and infection rates continue to rise (14, 15, 16, 17, 18, plus an additional reference)."
I respectfully suggest including the following citation:
Lane CR, Seemann T, Worth LJ, Easton M, Pitchers W, Wong J, Cameron D, Azzato F, Bartolo R, Mateevici C, Marshall C, Slavin MA, Howden BP, Williamson DA. Incursions of *Candida auris* into Australia, 2018. Emerg Infect Dis. 2020;26(6):1326-1328. doi: 10.3201/eid2606.190936.

Thank you for suggesting this citation and re-wording, which improve the text. We have also separated this sentence from the next for clarity. We have also now confirmed detection of *C. auris* in Antarctica, so we have re-phrased this sentence as per your recommendations and included a further reference and clarification as a result (now lines 58-59).

Line 65
Replace "uncommon" with "low", as percentages in the 0–9% range are more accurately characterised in this manner.

We have re-written this line accordingly (now lines 64-68).

Line 71
Replace "murine infections models" with "murine candidiasis model".

Done (now line 73).

Lines 81–83
The authors may wish to clarify whether the term "strains" or "clinical isolates" is most appropriate in the context of the mutations described.

We now use the term "clinical isolates" to refer to *C. auris* taken from patients and tested for elevated MICs (now line 85).

Lines 90–93
Replace "…has been reported in patients receiving treatment…" with "…has been reported in isolates from patients receiving treatment…" to maintain precision.

Done (now line 94-95).

Line 100
Replace with the fuller formulation:
"*Candida parapsilosis, Candida glabrata* (also named *Nakaseomyces glabratus*) and *C. albicans*. Heteroresistance…"

Done (now line 102-103).

Lines 113–115
Replace "*C. auris* bloodstream infections" with "*C. auris* candidaemia", consistent with the terminology used in Figure 1.

Done (now line 117).

Line 124
Replace “two patients with bloodstream infections (BSI)” with “two patients with candidaemia”.

Done (now line 127).

Line 128
Replace “initial BSI” with “initial candidaemia”.

Done (now line 131).

Line 133
Replace “the fungal infection” with “the candidiasis”.

Done – and for consistency with the above line replaced with “candidaemia” (now line 136-138).

Line 173
Replace “echinocandin sensitive” with “echinocandin susceptible”.

Done (now line 177).

Lines 302–303
Replace “echinocandin sensitivity” with “echinocandin susceptibility”.

Done (now line 324).

Lines 363–364
Replace “host immunosuppression” with “host immunodeficiency”, as critically ill patients may present with variable degrees of immune dysfunction, not necessarily resulting from immunosuppression.

Done – and following your logic, replaced with “host immune dysfunction” to make critical illness-associated immune dysfunction clear from acquired and intrinsic immunodeficiencies (now line 405).

Lines 371–372
Please include a link or bibliographic reference to the Wellcome Trust funded COMBAT *Candida* clinical trial.

This has now been registered: We have provided the UK Clinical Study Registry Code (ISRCTN: 56596656) in the main text (now lines 413-4).

Reviewer 2
The authors discuss possible mechanisms of echinocandin resistance beyond mutations in *FKSI/FKS2*, which is an interesting observation. However, the study used lab-derived large and small colonies exposed to high concentrations of echinocandins rather than patient isolates. It is also unclear whether the patients died because of echinocandin therapy failure or for other reasons, since *C. auris*

invasive infections usually occur in immunocompromised patients. The detail comments are as follows:

It is true that these patient-derived clinical isolates were grown in the laboratory during exposure to high anidulafungin concentrations. However, the choice of 8 µg/mL is one dilution above the CLSI breakpoint of 4µg/ml and reflects physiological concentrations identified in critically ill patients. Our study represented a single experimental passage rather than extended evolution, which is often used to interrogate pathways to resistance emergence. To address this comment we have now added several sentences to the Discussion to expand on the relevance of *in vitro* exposure levels to those seen *in vivo* in pharmacokinetic studies of anidulafungin in critically ill patients (see lines 347-352).

Additionally, we have now clarified that neither death was directly attributed to candidaemia (lines 137-138).

Specific comments:
* The authors jump between CLSI and EUCAST methods. They should specify why this is so as the breakpoints are very different between the two methods (CLSI resistance ≥4 µg/mL and EUCAST resistance >0.25 µg/mL).

Thank you for this important point, which we realise needs clearer explanation. At the time of both patients' bloodstream infections, clinical service related *C auris* isolate MIC testing was performed at the UKHSA Mycology reference laboratory in Bristol, which uses CLSI methodology for antifungal susceptibility testing, with the tentative breakpoint of ≥4 µg/mL recommended by CDC (https://www.cdc.gov/candida-auris/hcp/laboratories/antifungal-susceptibility-testing.html). We subsequently repeated the MIC testing, testing the parent 'clinical' isolate alongside the lab-evolved 'big' and 'small' colonial variants in our laboratory using the EUCAST method, which had no published breakpoints prior to the recent publication in 2025, using epidemiological cutoff values ECOFFs (R >0.25). It is beyond the scope of this body of work to distinguish the limitations of breakpoint determinations, which for CLSI are "based on the modal distribution of echinocandin MICs of approximately 100 isolates from diverse geographic locations" (as per link above), and for EUCAST are "determined combining visual inspection of aggregated MIC distribution (eyeball method)…" (https://pubmed.ncbi.nlm.nih.gov/40728839/). Instead, we chose to highlight the general limitations of apparently clear-cut breakpoints in the context of treatment failure and evidence of heteroresistance, with an exploration of genotypic and phenotypic mechanistic underpinning.

We have now clarified that CLSI testing was performed as part of routine microbiological testing with subsequent EUCAST testing of clinical and evolved isolates as part of our study in the Methods section (lines 432-434) and included further detail in the third paragraph of the Discussion (lines 352-362).

* In Figure 3F, the data might be better represented on a log scale as the time interval between tick marks varies greatly

Done.

* Line 63, also bring reference Zhu et al. reference 12 as Clade I and amphotericin resistance.

Done (now line 65).

* Line 70, add another reference by Zhu et al. as this talks about several mutations in the hotspot 1 leading to echinocandin resistance https://journals.asm.org/doi/10.1128/jcm.01767-22

Done (now lines 71-2).

* In line 194, the concentration for resistance to anidulafungin has the unit g/mL. It should be ug/mL

Done (now line 197).

* In the Discussion, starting at line 310, the authors propose a theory that given the high concentrations of AND that the patient was receiving, populations of SCV and LCV developed under the antifungal pressure and were able to escape this pressure with mutations in CRZ1. If this was indeed the case, then one would expect the original clinical isolate collected from the patient to exhibit multiple colony morphologies (SCV and LCV) as they say these are stably maintained when sub cultured on media lacking AND. In addition, if their theory was correct, I would also expect the original clinical strains to have mutations in *CRZ1*. However, based on the data presented in Figure 4, none of the original clinical isolates had any mutations in *CRZ1*.

The original clinical isolates were not screened directly for SCVs and LCVs because they were susceptible to drug, according to MIC testing. Also, at this point, we had not yet observed SCVs and LCVs, which were only observed later during our *in vitro* analyses of minority subpopulations representing the phenomenon of heteroresistance. *In vitro* anidulafungin exposure at supra-MIC concentration was required to observe these very small sub-populations, representing <0.001% of the total population (Figure 3A). These SCVs and LCVs may have been present in the initial clinical isolate, but they would not have been detected when cultured on media lacking anidulafungin and would have been missed by conventional MIC testing. Given the low abundance of these subpopulations, corresponding *CRZ1* mutations would not be detectable by whole genome sequencing (variant calling filtration typically requires a high allele frequency within a population and would not pick up alleles with <0.001% frequency by Illumina sequencing.) To address the Reviewer's point, we have added the following explanation to the text (lines 340-342): As standard variant calling methods exclude infrequent alleles, *CRZ1* escape mutations would be difficult to detect during the collection of clinical isolates given the low frequency of drug tolerant cells in heteroresistant isolates."

* The authors also suggest that ploidy may play a role in AND resistance, but do not demonstrate any difference in ploidy of one clinical strain from a control *C. auris* clade I strain. In Figure 6, the authors are comparing ploidy of one clinical isolate grown without AND, and the derived SCV and LCV grown with AND. The authors should include SCV and LCV ploidy when grown in the absence of AND, & if possible, the original clinical strain in the presence of AND. If the latter is possible, it would also be helpful to see if returning the culture to plates lacking AND leads to a decrease in ploidy again (which could support their hypothesis that ploidy was higher in the patient during AND treatment and went back down once the patient's isolate was cultured in the absence of drug).

Thank you for raising these points, which we are glad address with both clarification and additional experimental data. In our original submission, the ploidies of three Clade I isolates were compared with those of isolates from Clades II, III, IV and V (see Figure S4E). We suggested that ploidy may play a role in the development of anidulafungin resistance, not in anidulafungin resistance *per se*, and therefore, it is not clear to us why different isolates would be expected to display significant differences in their ploidies in the absence of drug pressure. After all, we observed heterogeneity in the ploidy of cell populations across all the isolates we examined. This heterogeneity yields subsets of cells with elevated ploidy (Figure S4E) that may promote the emergence of anidulafungin resistance under drug pressure.

The Reviewer then suggests that we compare the ploidies of parental, SCVs and LCVs in the presence and absence of drug. An important caveat is that parental isolates do not grow adequately in the presence of drug, unless they have developed an SCV or LCV phenotype. We performed additional experiments and now show that, in the absence of drug, the heterogeneous ploidies of variants rapidly revert to the haploid parental phenotype following growth in the absence of drug. These new data, which reinforce our suggestion that ploidy plays a role in the development of anidulafungin resistance (rather than conferring anidulafungin resistance) are now included in Figure 6D and described on lines 297-312 and 320-321.
